# A previously uncharacterized divisome-associated lipoprotein, DalA, is needed for normal cell division in *Rhodobacterales*

**DOI:** 10.1128/mbio.01203-23

**Published:** 2023-06-30

**Authors:** François Alberge, Bryan D. Lakey, Ryan E. Schaub, Alice C. Dohnalkova, Kimberley C. Lemmer, Joseph P. Dillard, Daniel R. Noguera, Timothy J. Donohue

**Affiliations:** ^1^Wisconsin Energy Institute, Great Lakes Bioenergy Research Center, University of Wisconsin-Madison, Madison, Wisconsin, USA; 2 Laboratory of Genetics, University of Wisconsin-Madison, Madison, Wisconsin, USA; 3 Department of Medical Microbiology and Immunology, University of Wisconsin-Madison, Madison, Wisconsin, USA; 4 Environmental Molecular Sciences Laboratory, Pacific Northwest National Laboratory, Richland, Washington, USA; 5 National Renewable Energy Laboratory, Golden, Colorado, USA; 6 Department of Civil and Environmental Engineering, University of Wisconsin-Madison, Madison, Wisconsin, USA; 7 Department of Bacteriology, University of Wisconsin-Madison, Madison, Wisconsin, USA; Max-Planck-Institut fur terrestrische Mikrobiologie, Marburg, Germany

**Keywords:** cell division, *Alphaproteobacteria*, peptidoglycan, cell envelope, lipoprotein

## Abstract

The bacterial cell envelope is a key subcellular compartment with important roles in antibiotic resistance, nutrient acquisition, and cell morphology. We seek to gain a better understanding of proteins that contribute to the function of the cell envelope in *Alphaproteobacteria*. Using *Rhodobacter sphaeroides*, we show that a previously uncharacterized protein, RSP_1200, is an outer membrane (OM) lipoprotein that non-covalently binds peptidoglycan (PG). Using a fluorescently tagged version of this protein, we find that RSP_1200 undergoes a dynamic repositioning during the cell cycle and is enriched at the septum during cell division. We show that the position of RSP_1200 mirrors the location of FtsZ rings, leading us to propose that RSP_1200 is a newly identified component of the *R. sphaeroides*’ divisome. Additional support for this hypothesis includes the co-precipitation of RSP_1200 with FtsZ, the Pal protein, and several predicted PG L,D-transpeptidases. We also find that a ∆*RSP_1200* mutation leads to defects in cell division, sensitivity to PG-active antibiotics, and results in the formation of OM protrusions at the septum during cell division. Based on these results, we propose to name RSP_1200 DalA (for division-associated lipoprotein A) and postulate that DalA serves as a scaffold to position or modulate the activity of PG transpeptidases that are needed to form envelope invaginations during cell division. We find that DalA homologs are present in members of the *Rhodobacterales* order within *Alphaproteobacteria*. Therefore, we propose that further analysis of this and related proteins will increase our understanding of the macromolecular machinery and proteins that participate in cell division in Gram-negative bacteria.

**IMPORTANCE:**

Multi-protein complexes of the bacterial cell envelope orchestrate key processes like growth, division, biofilm formation, antimicrobial resistance, and production of valuable compounds. The subunits of these protein complexes are well studied in some bacteria, and differences in their composition and function are linked to variations in cell envelope composition, shape, and proliferation. However, some envelope protein complex subunits have no known homologs across the bacterial phylogeny. We find that *Rhodobacter sphaeroides* RSP_1200 is a newly identified lipoprotein (DalA) and that loss of this protein causes defects in cell division and changes the sensitivity to compounds, affecting cell envelope synthesis and function. We find that DalA forms a complex with proteins needed for cell division, binds the cell envelope polymer peptidoglycan, and colocalizes with enzymes involved in the assembly of this macromolecule. The analysis of DalA provides new information on the cell division machinery in this and possibly other *Alphaproteobacteria.*

## INTRODUCTION

The bacterial cell envelope is an essential compartment that governs an organism’s shape, creates a physical barrier with the environment, and needs continual remodeling to ensure proper cell growth and division. In Gram-negative bacteria, the cell envelope is composed of the inner membrane (IM), the periplasm, the outer membrane (OM), and a peptidoglycan (PG) layer that is composed of a mesh of repeating N-acetyl glucosamine and N-acetyl muramic acid strands cross-linked by peptide stems. Because of the critical role the cell envelope plays in bacterial survival, we are interested in elucidating the proteins needed for cell envelope integrity and function, particularly in microorganisms that have poorly defined macromolecular machineries for cell envelop synthesis and cell division and are of potential industrial interest.

In bacteria, lipoproteins are anchored to a membrane by a lipid moiety, and many of them are important actors in cell envelope integrity. For instance, the OM lipoprotein Lpp is one of the most abundant proteins in *Escherichia coli*, and its covalent binding to PG links these two cell envelope components (1 - 3). The bacterial cell envelope is continuously remodeled throughout the division cycle. To maintain cell envelope integrity and viability, there needs to be tight synchronization of multi-protein complexes that span the IM, PG, and OM as cells progress through the elongation and division phases of the cycle (4). In *E. coli* and other bacteria, specific multi-protein complexes have been identified that function, respectively, in the elongation (elongasome) and division (divisome) phases of the cell cycle (5 - 8).

Analysis of these multi-protein complexes has demonstrated the presence and role of OM lipoproteins in both the elongasome and the divisome. Another OM lipoprotein, Pal, non-covalently binds PG and is important for normal cell division by controlling invagination of OM, PG, and IM during septation (9, 10). Furthermore, the *E. coli* lipoproteins LpoA and LpoB are part of the elongasome and divisome complexes, respectively, and control function of transpeptidases needed to coordinate PG synthesis (11, 12).

Unfortunately, much less is known about the role of lipoproteins in cell elongation and division of multi-protein complexes in other bacteria (13 - 16), including *Alphaproteobacteria*, where different modes of cell elongation, division, and positioning of the envelope multi-protein complexes are reported (17, 18). For example, a polar cell elongation is seen in *Rhizobiales* (19 - 21), budding in *Rhodobacterales* (22), and lateral elongation in *Caulobacterales* (23, 24). Identifying the multi-protein complexes and the mechanisms underlying these different modes of cell growth could reveal new targets for antibiotic development or inform strategies for bioproduct secretion.

We are studying cell envelope functions in *Rhodobacter sphaeroides*, a rod-shaped *Alphaproteobacteria* belonging to the *Rhodobacterales* order. The *R. sphaeroides* elongasome subunit MreB exhibits an atypical position at the midcell for most of the cell cycle (25) compared to the known heterogeneous localization of MreB during cell elongation in *E. coli* (26). In addition, there are no known homologs of the Lpp and LpoA/LpoB lipoproteins in *R. sphaeroides* and other *Alphaproteobacteria*, suggesting differences in the process and control of cell elongation and division in *R. sphaeroides* compared to other Gram-negative bacteria that also divide by symmetric fission.

Indeed, in recent work, we reported that the previously uncharacterized two-component systems CenKR and NtrYX coordinate cell elongation and division in *R. sphaeroides* and other *Alphaproteobacteria* (27 - 29). CenKR is an essential two-component system and directly controls transcription of *RSP_1200*, a previously uncharacterized gene that, when disrupted, led to the production of extracellular lipid vesicles and a higher sensitivity to chemicals that act at the cell envelope (30). In this work, we show that *RSP_1200* encodes an ≈18 kDa lipoprotein that can non-covalently bind PG. Like many lipoproteins in other Gram-negative bacteria, RSP_1200 is localized to the OM (31, 32). Furthermore, RSP_1200 forms a complex with proteins known or predicted to be subunits of the divisome in Gram-negative bacteria, including Pal, FtsZ, and PG maintenance enzymes (5, 33). We find that cells lacking *RSP_1200* exhibit increased sensitivity to compounds that target PG biogenesis, have defects in cell division, and form OM protrusions at the septum. Based on our findings, we propose that RSP_1200 is a newly discovered modulator of PG transpeptidases that are needed for elongation and to form envelope invaginations during cell division in *R. sphaeroides* and possibly other *Rhodobacterales* that contain homologs of this OM lipoprotein. Because of its predicted role in cell division, we refer to RSP_1200 as division-associated lipoprotein A (DalA) throughout the remainder of this paper.

## RESULTS

### DalA is an OM lipoprotein that binds to subunits of the bacterial divisome

**Fig 1:**
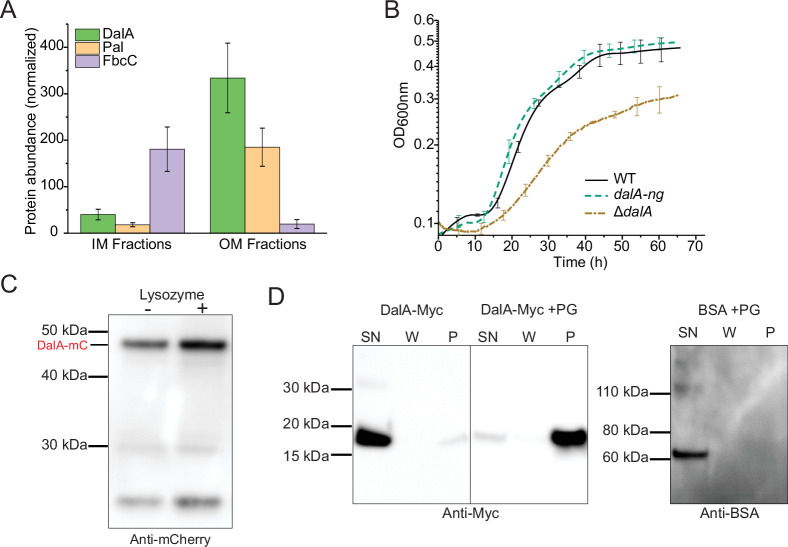
DalA is a PG-associated OM lipoprotein. (**A**) *R. sphaeroides* strain 2.4.1 membranes were separated on a sucrose gradient, and the proteins in each fraction were analyzed by mass spectrometry. (**B**) Growth curves from four independent cultures grown at 30°C. (**C**) Western blot analysis of lysates of *dalA-mCherry* cultures before and after lysozyme treatment using antibodies against mCherry. Bands ≤30 kDa correspond to mCherry degradation products. (**D**) Western blot analysis to test *in vitro* binding of DalA or bovine serum albumin (BSA) with PG. As described in Materials and Methods, the supernatant fractions (SN), wash fractions (W), and pellet fractions (P) containing the PG were analyzed for protein content.

Based on its amino acid sequence, we predict that the *dalA* gene product is an OM lipoprotein because it contains an N-terminal signal peptide with a cysteine residue following a typical signal peptidase II cleavage site (Fig. S1), features that characterize OM lipoproteins (34). To test the cellular location of DalA, we fractionated *R. sphaeroides* cells and monitored the abundance of this protein in IM and OM by performing an liquid chromatography with tandem mass spectrometry (LC-MS/MS) analysis of peptides that were derived from proteins in these fractions (Fig. 1A). To assess the effectiveness of the IM and OM separation, the enrichment in peptides of the OM Pal lipoprotein and of the IM FbcC subunit of the cytochrome *c*_1_ subunit of the cytochrome *cb*_1_ complex was measured. We found that peptides derived from DalA and Pal were enriched in purified OM fractions, while isolated IM fractions were enriched with peptides of FbcC. From this, we conclude that DalA is localized to the OM.

To further evaluate the DalA localization, we fused the fluorophore mNeonGreen (NG) to the C-terminus of the gene and recombined the *dalA-ng* fusion into the native *dalA* locus of the *R. sphaeroides* genome. When we analyzed the function of the DalA-NG fusion protein by testing growth (Fig. 1B), we found no significant difference in the generation time or final cell density between cells containing the DalA-NG fusion protein and one containing the wild-type (WT) version of this protein. To test if DalA interacted with other proteins, we added antibodies against NG to precipitate proteins from extracts of cells containing the DalA-NG fusion. Mass spectrometry of peptides derived from the resulting immunoprecipitation identified DalA and proteins involved in PG assembly, including homologs of L,D-transpeptidases (LDTs) (RSP_1199, RSP_0243) plus other PG-associated enzymes (Table 1). These immunoprecipitates also contained the *R. sphaeroides* homolog of Pal (RSP_0668), which links PG to the OM in other bacteria, and FtsZ (RSP_2114), which is part of the divisome in this (35) and other bacteria (10). From these results, we conclude that DalA is an OM lipoprotein that interacts with envelope-associated PG assembly proteins and subunits of the divisome .

**TABLE 1:** Top cell envelope proteins identified by mass spectroscopy co-precipitated with DalA-NG^*a*^

Protein	Name	Accession number	Protein family	Predicted localization
RSP_1440		Q3J6K3	TonB-dependent, hydroxamate-type ferric siderophore, OM receptor	OM
RSP_0842		Q3IZL2	Putative porin	OM
RSP_1880		Q3J5A5	PG-binding domain-containing protein	P/OM
RSP_0251		Q3J1B1	OM efflux protein	OM
RSP_1033		Q3IZ17	OmpA family protein	OM
RSP_2400		Q3J3S3	Spermidine/putrescine import ATP-binding protein	IM
RSP_0240	PntA	Q3J1C2	NAD(P) transhydrogenase subunit alpha	IM
RSP_1199		Q3IYK3	YkuD domain-containing protein (predicted LDT)	P/OM
RSP_2299		Q3J431	ATP synthase subunit beta 1	IM
RSP_0668	Pal	Q3J042	PG-associated protein	OM
RSP_0665	FtsH	Q3J045	ATP-dependent zinc metalloprotease FtsH	IM
RSP_2711	BamA	Q3J2W8	OM protein assembly factor BamA	OM
RSP_2855		Q3J2M9	Cation/multi-drug efflux pump	IM
RSP_2465	MltG	Q3J3L0	Endolytic murein transglycosylase (PG polymerization terminase)	P/OM
RSP_2402		Q3J3S1	TonB-dependent Vitamin B12 OM receptor	OM
RSP_1169	SecA	Q3IYN1	Protein translocase subunit SecA	IM
RSP_0243		Q3J1B9	Putative lipoprotein (predicted LDT)	P/OM
RSP_1183		Q3IYL7	Uncharacterized protein	IM
RSP_0357		Q3J103	Protein HflK	IM
RSP_1882	PotA	Q3J5A4	Spermidine/putrescine import ATP-binding protein PotA	IM
RSP_2114	FtsZ	Q3J4L5	Cell division protein FtsZ	IM

^*a*^ Table regrouping the proteins associated with DalA-NG during the co-immunoprecipitation and identified by nano LC-MS/MS. The table excludes the identified cytoplasmic proteins and only shows the proteins known to be associated either with the IM, the OM or present in the periplasm (P). Proteins are sorted by their total spectrum count in the co-immunoprecipitation.

### DalA binds PG

The proteins present in these immunoprecipitates led us to consider whether DalA could bind PG. To test this hypothesis, we monitored the behavior of DalA on denaturing gels after crude extracts from cells harboring a functional mCherry-tagged protein (Fig. S2A) were incubated in the absence or presence of lysozyme, which is known to cleave glycan chains of PG (36). We chose to use crude extracts from this strain since the antibodies to the mCherry domain were of a sensitivity needed to detect complexes via Western blots. If DalA was covalently bound to PG, we predicted there will be a difference in the migration of DalA between extracts that were (+) and were not (−) treated with lysozyme. We found that the migration of the DalA protein was the same in both lysozyme-treated and untreated samples (Fig. 1C), leading us to conclude that this protein is not covalently bound to PG.

To test if DalA could bind PG non-covalently, we incubated either a purified mCherry or a Myc-tagged version of DalA with PG that was isolated from *R. sphaeroides*. After incubating purified DalA fusion proteins with isolated PG samples, the mixture was centrifuged to collect the PG, the pellet was washed, and analyzed by Western blot using tag-specific antibodies to test for the presence of the DalA protein. We found that either DalA-Myc or DalA-mCherry was present in the PG-containing pellet after centrifugation (Fig. 1D; Fig. S2B). As a control, we performed PG-binding assays in which bovine serum albumin (BSA) was used instead of DalA and did not detect this protein in the PG-containing pellet (Fig. 1D). Combined, these observations support the hypothesis that DalA can bind PG non-covalently.

### The dynamic envelope localization of DalA during the cell cycle

**Fig 2:**
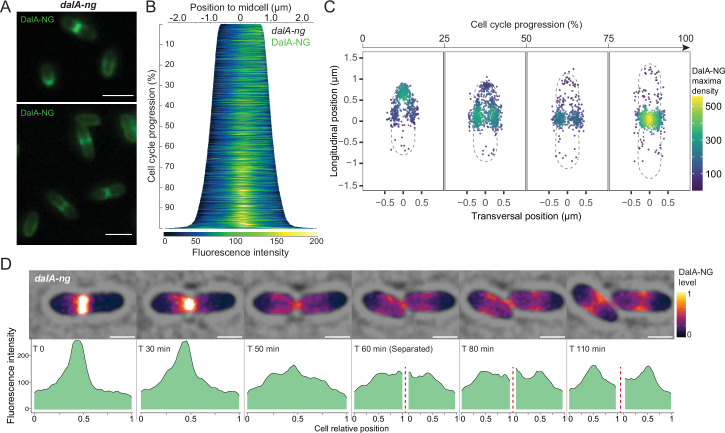
DalA movement during the cell cycle. Cultures containing *dalA-ng* fusion were imaged during exponential growth. (**A**) Fluorescence microscopy images of DalA-NG in cells. Scale bars represent 2 µm. (**B**) Demograph depicting DalA-NG fluorescence profiles after cells were sorted from top to bottom by length and laterally to place the pole with the most fluorescence on the right. (**C**) Density map of DalA-NG maxima during cell cycle after cells were equally separated by length in four groups (from shortest on the left to longest on the right). The gray line represents the average cell shape of each group. (**D**) Pictures of a time-lapse recording set with a 10-min interval of a dividing *dalA-ng* cell, the brightfield, and DalA-NG fluorescence signal (plasma scale) were merged. Cell fluorescence profiles are provided below per normalized length. Scale bars represent 1 µm.

Given the presence of typical divisome components in the proteins that co-immunoprecipitated with DalA (e.g., Pal and FtsZ), we monitored by fluorescence microscopy the subcellular position of a DalA-NG fusion during cell growth. We found that most cells containing the DalA-NG fusion showed the most intense fluorescent foci at midcell. This analysis led us to propose there was an enrichment of DalA at the septum, as would be expected if this protein was associated with the divisome (Fig. 2A).

It is known that major subunits of the divisome (Pal, FtsZ, etc.) undergo cell cycle-dependent changes in their envelope localization in other bacteria (19, 37 - 40). To test if there are changes in the envelope position of DalA as cells progress through the cycle, we generated a demograph of the DalA-NG fluorescence profiles in exponentially growing cells that were sorted by length (Fig. 2B). In growing cells, cell cycle progression directly correlates with cell length, allowing the description of the protein spatio-temporal dynamics (37, 40, 41). This analysis revealed that, in shorter cells (top cells of the demograph, until ≈15% of cycle progression), which likely represent those that recently completed division, DalA-NG is localized predominantly at one of the two poles and moves to the midcell as cells get longer (Fig. 2B). The predominant midcell localization of DalA-NG was observed early in the cycle (≈30% of cell cycle progression) and increased until the cycle completion. By projecting DalA-NG fluorescence maxima on cells as they progress through the division cycle (Fig. 2C), we find that, at later division stages, DalA moves to the site of cell constriction and is ultimately concentrated at the septum. These data provide evidence for differences in the envelope position of DalA as cells progress through the cell cycle.

We also used data from time-lapse experiments that follow DalA-NG fluorescence in individual cells to test our hypothesis that this protein was enriched at the newly formed pole of daughter cells immediately after division (Fig. 2D; Fig. S3A). To do this, we monitored cells undergoing division and analyzed the fluorescence profiles of daughter cells after their separation in order to distinguish new and old poles (Fig. S3B). This analysis revealed that a higher DalA-NG fluorescence is observed at the new cell pole. We also used these data to calculate the ratio of the DalA fluorescence intensity in the new pole divided by the old pole for each cell. This analysis found that, for all the cells, the fluorescence ratio was greater than 1 in the new pole, with a median ratio of 3 for fluorescence intensity at the new cell pole (Fig. S3C). During cell separation, we also observed a redistribution of DalA from the septum (future new pole of daughter cells) to the midcell of daughter cells (Fig. 2D), consistent with the previous analyses that showed a similar localization and redistribution pattern of this protein at the beginning of the cycle (Fig. 2B and C). This is additional evidence in support of our hypothesis that in the newly divided cells, DalA is present mostly at the new pole, then moves and becomes enriched at the septum prior to cell division, and remains there until the two daughter cells are formed. Thus, we propose that DalA exhibits spatio-temporal changes in envelope position as cells proceed through the cell cycle.

### DalA is associated with the PG synthesis machinery and the Pal protein

**Fig 3:**
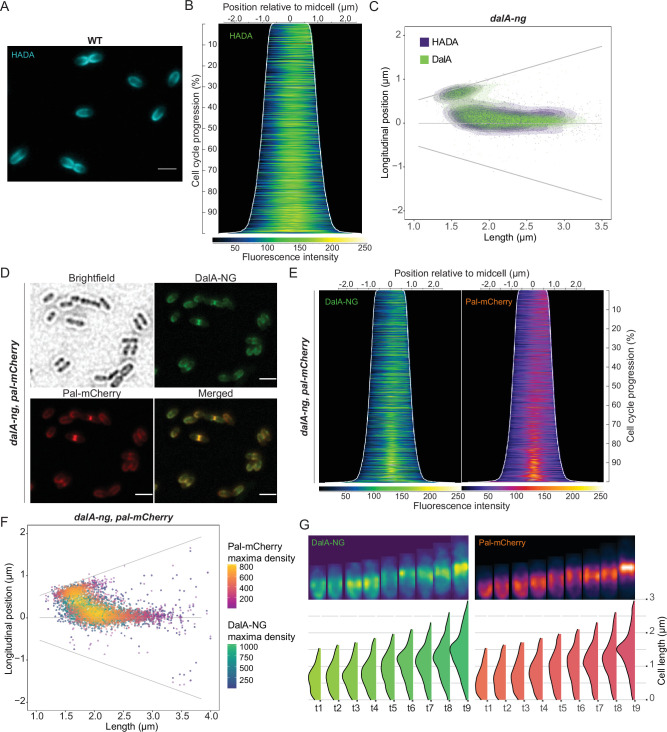
Localization of Pal and PG remodeling. (**A**) A WT culture was treated with HADA during exponential growth and imaged by fluorescence microscopy. (**B**) Corresponding demograph of HADA-stained WT cells. HADA fluorescence profiles were sorted longitudinally by cell length and laterally to display the most intense pole on the right. (**C**) Heatmap representing longitudinal localization of fluorescence maxima for *dalA-ng* strains stained with HADA over cell length, for both DalA-NG (green) and HADA (blue) fluorescence. (**D**) Micrographs of a strain containing both *pal-mCherry* and *dalA-ng* constructions during exponential growth. (**E**) Corresponding demograph of cells containing both *pal-mCherry/dalA-ng* fusions after fluorescence profiles were sorted longitudinally by cell length and laterally to place the pole with the most intense DalA-NG fluorescence on the right. (**F**) Density heatmap of longitudinal colocalization of detected fluorescence maxima over cell length for DalA-NG (blue to green scale) and Pal-mCherry (purple to yellow scale). (**G**) Time-lapse fluorescence microscopy of a cell containing *dalA-ng* and *pal-mCherry* (top). Each frame is taken over a 45-min interval during exponential growth. The fluorescence profiles for each frame and channel were computed (bottom). Scale bars represent 2 µm.

Since DalA can bind PG and form a complex with several PG modification enzymes, we wished to compare DalA position in the envelope to that of the *R. sphaeroides* PG synthesis machinery. To do this, we used HADA, a fluorescent D-amino acid (FDAA) that is incorporated in PG peptides, to visualize zones of synthesis of this cell wall polymer (24). We found that HADA labeling in *R. sphaeroides* initiates in the new poles of daughter cells before it moves toward the midcell, mirroring the previously observed positioning of DalA as the cell progresses through the division cycle (Fig. 3A and B). When we added HADA to a *dalA-ng* strain, we found an overlap in the fluorescence formed by the two reporter molecules (Fig. S4). To support the predicted colocalization of these two signals along the cell cycle, we found that a heatmap of maxima localization in cells related to their length (Fig. 3C) showed a colocalization of DalA and HADA fluorescence during all phases of the cell cycle.

In *E. coli* and *Alphaproteobacteria*, the Pal protein is an important divisome subunit that both coordinates OM constriction and links the Tol-Pal complex to PG (10, 42 - 45). To monitor the cell cycle positioning of Pal and DalA, we analyzed *R. sphaeroides* cells containing both C-terminal Pal-mCherry fusion and DalA-NG fusion. This analysis revealed that Pal and DalA have similar envelope positions as cells progress through the division cycle (Fig. 3D through F), with a high level of correlation between the location of the two proteins (Fig. S4). In addition, time-lapse imaging of the DalA-NG and Pal-mCherry proteins in single cells showed that Pal and DalA are both enriched at the new pole of daughter cells before they migrate to the septum during division (Fig. 3G). Combined, these data indicate there is a high level of spatio-temporal colocalization of PG synthesis, Pal, and DalA during the *R. sphaeroides* cell cycle.

### DalA and Pal are each important for normal cell division

**Fig 4:**
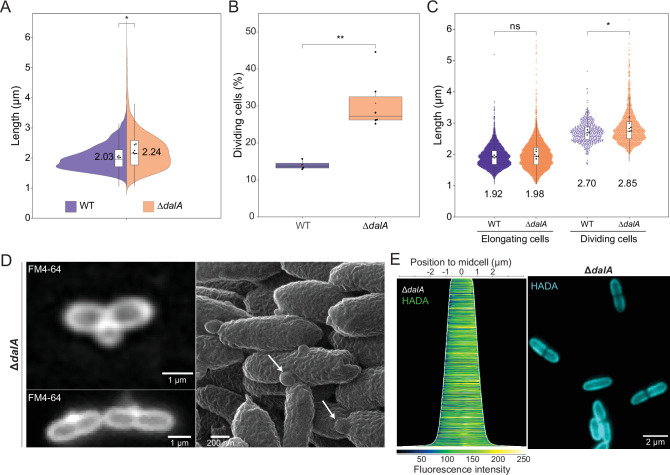
Analysis of cell division in a Δ*dalA* strain. (**A**) Distribution of cell lengths represented by a two-sided violin plot for WT cells (purple, left) and a Δ*dal*A strain (orange, right). Box plots are present, line represents the median, and box extends to the 25th and the 75th percentiles, whiskers indicate 1.5× interquartile range. Black dots represent the average cell length from each of at least four independent experiments. Global length average is indicated by text next to the box in micrometer. (**B**) Box plots showing the percentage of cells in division during exponential growth after analysis of WT and Δ*dalA* cells. Black dots represent the average percentage of each independent experiment, the red dot is the global average. (**C**) Violin plots of cell lengths by cell cycle stage. Black dots in box plots represent the mean length for each biological replicate, the number on top of each plot is the global average of length (µm). (**D**) Fluorescence images of Δ*dalA* cells stained with FM4-64 (left), helium-ion microscopy image of the same strain (right) showing OM protrusions (white arrows). (**E**) Fluorescence image of Δ*dalA* cells treated with HADA (right) and demograph of the HADA fluorescence cell profiles sorted longitudinally by length and laterally to display more intense pole on the right, obtained from three biological replicates (left). *P*-values were computed from mean values of biological replicates by unpaired two-tailed *t*-tests except for (**B**) where Mann-Whitney test was used (*, *P* ≤ 0.05; **, *P ≤* 0.01).

A Δ*dalA* strain grows slower than WT cells (Fig. 1B), so we compared the cellular architecture of WT and Δ*dalA* cells. We found that Δ*dalA* cells (2.24 µm) were longer than their WT counterparts (2.03 µm) (Fig. 4A). One explanation for the increased length of Δ*dalA* cells is that there is a larger fraction of dividing cells in the absence of DalA. To test this hypothesis, we exposed growing cells to the membrane dye FM4-64 and counted the number of Δ*dalA* and WT cells that contained a septum. This analysis showed that the Δ*dalA* culture had over twice as many cells that contain a septum (31%) than the WT population (14%) (Fig. 4B). However, when we analyzed the length of cells that are in the elongation phase of the cycle (those that lack a septum), we saw no significant difference in the length of the Δ*dalA* and WT cells (Fig. 4C). Combined, these data indicate that there is no significant effect of the loss of DalA during cell elongation and that the increased length of Δ*dalA* cells (Fig. 4A) reflects the presence of a larger number of dividing cells in the mutant population.

Previous work reported that cells containing a transposon insertion in *dalA* (RSP_1200) produced extracellular lipid vesicles (30). We found that both fluorescence microscopy of Δ*dalA* cells treated with FM4-64 and high-resolution technique helium ion microscopy revealed evidence for OM protrusions (Fig. 4D) from the septum region in ≈22% of the dividing cells (≈7% of total Δ*dalA* cells). In contrast, we failed to observe the formation of OM protrusions in WT cells by these techniques(Fig. S5).

To further test the role of DalA on PG synthesis, we used HADA to analyze this in Δ*dalA* cells. Unlike the situation in WT cells (Fig. 3A through C), the HADA fluorescence was distributed throughout the length of Δ*dalA* cells (Fig. 4E). Furthermore, for the longer Δ*dalA* cells, there was a low level of HADA staining at the septum (Fig. 4E), leading us to propose that the absence of DalA impacts the spatio-temporal movement of PG biosynthetic enzymes at the time of division.

**Fig 5:**
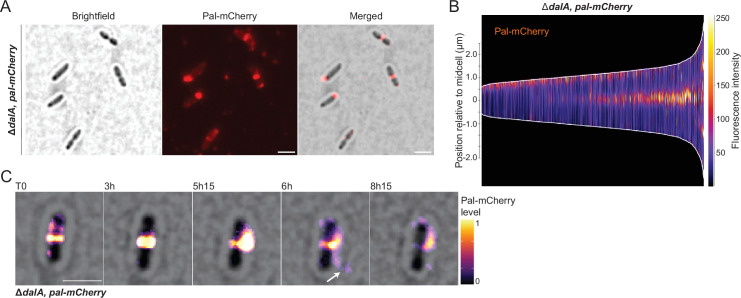
Δ*dalA* protrusions in Δ*pal* cells. (**A**) Microscopy image of the Δ*dalA* cells containing a *pal-mCherry* fusion and the corresponding demograph (**B**). Cell intensity profiles of Pal-mCherry fluorescence in Δ*dalA* strain were sorted longitudinally by length and laterally to display more intense pole on the right. (**C**) Time-lapse recording of a Δ*dalA* cell with brightfield channel and *pal-mCherry* fluorescence merged. Scale bars represent 2 µm.

To test if the position of Pal was affected in the Δ*dalA* strain, we analyzed the localization of this protein by recombining a *pal-mCherry* fusion gene in its native locus. We found that the Pal protein was present in distinct and more intense foci in the Δ*dalA* mutant than in WT cells (Fig. 5A and B). In contrast to the pattern of spatio-temporal positioning of Pal that was observed in WT cells (Fig. 3D through G), there were Pal foci in the Δ*dalA* mutant at both the new pole and the septum during elongation before it was localized to the septum during division (Fig. 5A and B). Time-lapse imaging Pal-mCherry localization in the Δ*dalA* mutant revealed the presence of fluorescent foci at the septum, and in some Δ*dalA* cells, there was evidence for formation of membrane protrusions at the division septum (Fig. 5C), reminiscent of those seen by staining with FM4-64 or by helium ion microscopy of cells lacking DalA (Fig. 4D). Indeed, time-lapse analysis of the Δ*dalA* mutant provides evidence for the release of membrane protrusions that contain high level of Pal-mCherry into the medium (Fig. 5C). Combined, these results support the hypothesis that Pal and DalA form a complex that is required for normal cell division by *R. sphaeroides*.

**Fig 6:**
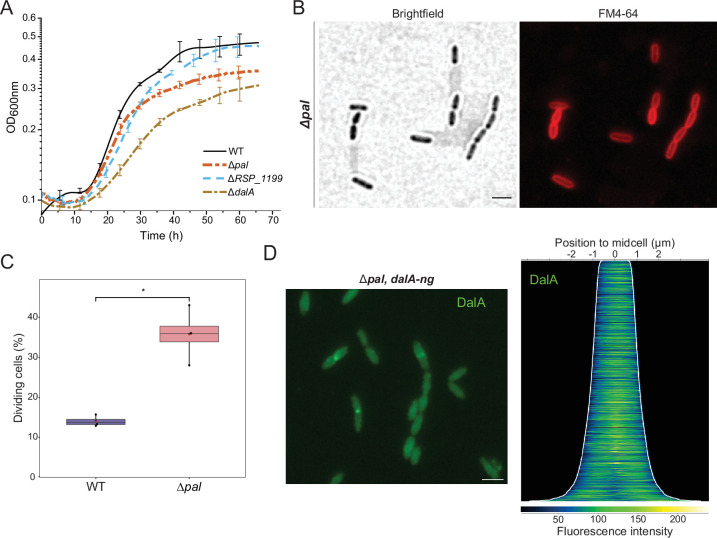
Δ*pal* affects DalA localization. (**A**) Growth curves obtained from four biological replicates of cultures incubated at 30°C. (**B**) Microscopy analysis of a Δ*pal* strain during exponential growth with membrane dye FM4-64 added before visualization. (**C**) Box plots showing the percentage of cells in division. Black dots represent the average percentage of each independent experiment, the red dot is the global average. *P*-value was computed from mean values of biological replicates by unpaired Mann-Whitney (*, P ≤ 0.05). (**D**) Fluorescence signal of DalA-NG in the Δ*pal, dalA-ng* strain (left), and the corresponding demograph (right). Fluorescence cell profiles were sorted longitudinally by length and laterally to display more intense pole on the right, obtained from three independent replicates. Scale bars represent 2 µm.

Defects in cell division and formation of membrane protrusions at the septum are known phenotypes of *E. coli tol-pal* mutants (10), so we wanted to test if and how loss of Pal impacted *R. sphaeroides*. We found that a Δ*pal* mutant has an increased generation time compared to WT cells (22% ± 4% increased generation time), but it grows faster than a Δ*dalA* strain (52% ± 8% increased generation time) (Fig. 6A). As with the Δ*dalA* mutant, there is an increase in Δ*pal* cells in the division phase of the cell cycle compared to WT culture (36% ± 5% of dividing Δ*pal* cells compared to 14% ± 1% for WT cells) (Fig. 6B and C). There was also evidence for cell chaining (>2 cells joined together, 4% of total cells) in the Δ*pal* mutant that was not observed in either WT or Δ*dalA* cells. However, there was no sign of OM protrusions from the septum or elsewhere along the cell surface in the Δ*pal* mutant (Fig. 6B).

When we placed a DalA-NG fusion in the chromosome of the Δ*pal* mutant, we observed modifications in pattern of DalA subcellular positioning (Fig. 6D). In the absence of Pal, the DalA-NG fluorescence is distributed throughout the cell before division, and there is less enrichment of the DalA-NG fusion at the septum during division than in WT cells. Western blot analysis with NG antibodies indicated there was approximately threefold decrease in DalA-NG abundance in the Δ*pal* strain than in WT cells (Fig. S6A). To test whether the change in DalA-NG envelope localization could be due to lower abundance of this fusion protein in the Pal mutant, we also analyzed a strain carrying the *dalA-ng* gene under the control of its own promoter on a plasmid. In a Δ*pal* strain containing this plasmid, we failed to observe the spatio-temporal positioning of DalA seen in WT cells, even though DalA-NG levels (as measured by Western blotting) were comparable or even slightly higher than those in WT cells (Fig. S6A and B). Thus, we conclude that the Pal protein is important to the observed changes in subcellular location of DalA seen in WT cells.

### FtsZ, DalA, and PG assembly enzymes are part of a *R. sphaeroides* divisome complex

**Fig 7:**
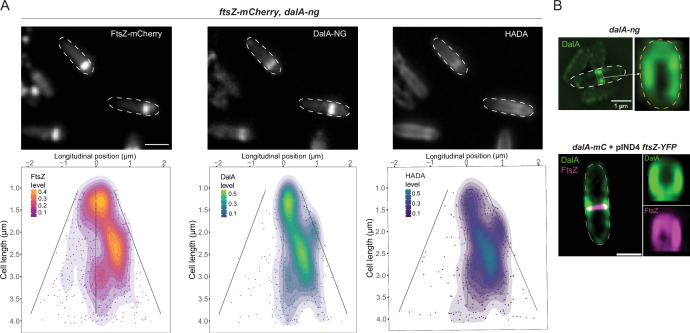
FtsZ rings colocalize with DalA. (**A**) Cells containing *fsZ-mCherry* and *dalA-ng* fusions were imaged (top). HADA was added during exponential growth. Maxima of fluorescence for each channel was identified and their longitudinal position and density plotted against the cell length (bottom). Cells were aligned to display the pole with the most intense FtsZ-mCherry fluorescence on the right. Analysis was done with data from three independent cultures. Scale bar represents 2 µm. (**B**) Three-dimensional reconstructed structured illumination microscopy image of cells containing a *dalA-ng* fusion (top) and *dalA-mCherry* cells (signal colored in green) containing *ftsZ-YFP* on plasmid (signal colored in magenta, bottom). A 10 µM IPTG was used to express *ftsZ-YFP*. Transversal volumetric renderings of the rings were obtained using clear volume (right of cell pictures).

FtsZ is a tubulin-like protein that helps recruit proteins to assemble a divisome ring structure at the midcell in many bacteria (8, 37, 46). The localization of DalA at the midcell during cell division and its co-immunoprecipitation with FtsZ prompted us to test for *in vivo* colocalization of these proteins. To do this, we created a strain containing both *dalA-ng* and *ftsZ-mCherry* fusions in the chromosome under control of their native promoters. We found that the presence of the FtsZ-mCherry protein led to some defects in cell shape and growth rate, plus the presence of FtsZ rings at positions other than midcell (Fig. 7A; Fig. S7A). Using the distribution of fluorescence maxima to monitor ring localization, we found that the DalA-NG protein was colocalized with FtsZ-mCherry (Fig. 7A; Fig. S4) even when FtsZ rings were mis-localized, suggesting that the cellular position of DalA is linked to the location of FtsZ rings. In addition, after treating cells containing the FtsZ-mCherry fusion with HADA, we observed colocalization of this fluorescent reporter of PG remodeling, FtsZ, and DalA. From this, we conclude that the FtsZ, DalA, and the PG assembly machinery are all part of a *R. sphaeroides* divisome.

To evaluate the organization of DalA and FtsZ at midcell during division, we used three-dimensional (3D) structured illumination microscopy (SIM) super-resolution microscopy of cells containing the DalA-NG fusion. Analysis of the fluorescence revealed that DalA-NG forms a ring-like structure following constriction of the division septum (Fig. 7B), similar to the behavior of the FtsZ protein in *E. coli* cells (47). Furthermore, we used cells containing a genomic *dalA-mCherry* fusion, displaying an identical phenotype as the ones expressing *dalA-ng* (Fig. S2A, S7B), associated to a plasmid containing a *ftsZ-YFP* gene described previously (35). It allowed us to observe DalA and FtsZ as part of putative constriction rings at the midcell (Fig. 7B). Combined, these findings predict that DalA plays a role in the *R. sphaeroides* cell cycle, possibly by participating in septum formation in association with FtsZ and other components of the divisome.

### DalA impacts PG composition

Based on the defects observed in the absence of DalA and its co-precipitation with predicted LDTs, enzymes that generate cross-links between the glycan chains or between PG and OM proteins, we hypothesize that the absence of DalA could negatively affect the degree of PG cross-linkage. To evaluate this hypothesis, we analyzed the composition of PG fragments in WT cells and those lacking *dalA*. We also analyzed the PG composition of WT cells grown in the presence of CuCl_2_, at concentrations known to inhibit the action of LDTs in *E. coli* and negatively affect the cross-linking of this cell wall polymer (48).

As expected, we were not able to detect any tri-tri cross-links between meso-diaminopimelic acid dimers (mDAP-mDAP linkage) when the cells are grown in the presence of CuCl_2_ (Table 2; Fig. S8). Tri-tri cross-links are specifically generated by LDTs (49), so their abundance can be used to test if the loss of DalA affects LDT activity. We observed an almost twofold decrease in the abundance of tri-tri cross-links in the *dalA* mutant (≈9.4%) compared to WT cells (≈18.4%) (Table 2; Fig. S8 ). This finding demonstrates the impact of a loss of *dalA* on PG composition, possibly due to a loss of an interaction of the OM lipoprotein with LDTs and other enzymes needed to remodel PG. In contrast, we found there was an increase in the level of tri-tri cross-links when *pal* is deleted (34.3% compared to 18.0% in WT cells), indicating that loss of different potential components of the cell wall assembly machinery does not exert the same impact on the pattern of PG cross-linking. We also found a decrease of Tri-Lys-Arg in the Δ*dalA* mutant compared to WT cells (4.2% versus 1.1%), providing further evidence for a role of DalA in the normal association of PG to one or more OM proteins. Further investigation is needed, but it could indicate that DalA plays a role in the regulation of physical associations between OM proteins and PG.

**TABLE 2:** Analysis of PG composition^*a*^

	Strain			
	WT	Δ*dalA*	Δ*pal*	WT + CuCl_2_
PG cross-link rate (%)	67.0 (±9.9)	61.9 (±7.5)	78.9 (±3.5)	33.0 (±4.8)
Tri-tri (LDTs) cross-links (%)	18.0 (±6.1)	9.4 (±2.4)	34.3 (±1.9)	ND
Tri-tetra cross-links (%)	18.3 (±3.2)	16.5 (±0.7)	27.8 (±4.2)	2.1 (±1.0)
Tetra-tetra cross-links (%)	14.8 (±5.5)	11.0 (±1.3)	6.0 (±1.3)	23.5 (±3.0)
Tri-Lys-Arg (%)	4.3 (±2.9)	1.1 (±0.6)	4.8 (±2.1)	4.9 (±1.9)

^*a*^ Quantification of the degree of PG cross-linkage, tri-tri cross-links (generated by LDTs), tri-tetra (LDTs and D,D transpeptidases), tetra-tetra (D,D transpeptidases), and Tri-Lys-Arg (OM-PG cross-links, LDTs) in *R. sphaeroides* strains. ND, none detected.

### Sensitivity of mutants lacking DalA and DalA-binding proteins to cell wall-active compounds

It has been shown that cells containing a Tn5 insertion in *dalA* have increased sensitivity to membrane- and envelope-active compounds, including inhibitors of PG transpeptidases (aztreonam) and detergents (SDS) (30). Based on this information and the observed cell cycle repositioning of DalA with subunits of the divisome, we tested the sensitivity of cells lacking LDTs predicted to bind DalA (RSP_1199, RSP_0243) to cell wall-active compounds (Table 1). We also tested the loss of *RSP_1201*, a gene downstream of *dalA* that encodes a predicted LDT but did not immunoprecipitate with DalA, compared to that of mutants lacking genes predicted to encode other LDTs.

**Fig 8:**
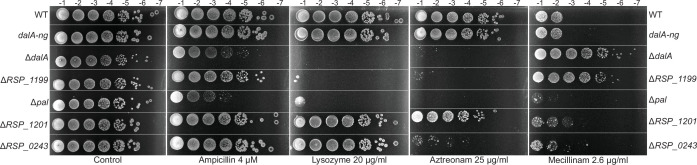
Sensitivity profiles of Δ*dalA* and Δ*RSP_1199* cells. Spot dilution assay. Strains were grown to exponential phase, diluted to an optical density of 0.5 at 600 nm, and spotted on Sistrom plates containing the indicated compounds as 10 µL of 10^−1^ to 10^−7^ dilutions.

We found that cells containing an in-frame deletion of *dalA* had increased sensitivity to compounds that interfere with PG cross-linking (aztreonam and ampicillin) compared to WT cells or a strain containing the DalA-NG fusion (Fig. 8). In addition, the Δ*dalA* mutant showed increased sensitivity to lysozyme compared to cells containing a functional DalA protein (Fig. 8). We also observed increased sensitivity of a Δ*pal* mutant to these compounds when compared to WT cells, consistent with the presence of these two OM lipoproteins in a predicted divisome.

We also tested the sensitivity of several strains to aztreonam, ampicillin, or lysozyme (Fig. 8). We found that cells lacking the predicted LDT RSP_1199 exhibited increased sensitivity to aztreonam and lysozyme, similar to what we found for the DalA and Pal mutants, while cells lacking the predicted LDT RSP_0243 only exhibited an increased sensitivity to aztreonam when compared to WT cells (Fig. 8). In addition, cells lacking RSP_1201 exhibited a lower level of sensitivity to aztreonam than any of the other mutants we tested, and they were resistant to concentrations of ampicillin that inhibited growth of the DalA and Pal mutants. These results suggest that loss of each of these predicted LDTs produces different levels of sensitivity to these known inhibitors of PG synthesis.

In addition, we analyzed sensitivity to each of these mutants to mecillinam, a known inhibitor of *E. coli* penicillin-binding protein 2 activity (50, 51). We found that a *R. sphaeroides* Δ*pal* strain showed increased sensitivity to mecillinam compared to WT cells (Fig. 8) as is found for Pal mutants of other bacteria (52). However, cells lacking either *dalA* or *RSP_1199* show increased resistance to mecillinam compared to WT cells (Fig. 8), suggesting a role for these two proteins in PG assembly.

## DISCUSSION

The envelope multi-protein complexes orchestrating cell elongation and division must be tightly linked to coordinate a myriad of events in the division cycle. Given the numerous roles of the cell envelope, it is important to understand the strategies used to coordinate elongation and division in bacterial species of biomedical, agricultural, environmental, and industrial importance. This work sought to gain a better understanding of the proteins coordinating cell envelope processes in *R. sphaeroides*. Previous work has shown that this rod-shaped bacterium divides by symmetric fission, but it lacks homologs of many OM lipoproteins known to orchestrate cell elongation and division in *E. coli,* a model organism that also divides by symmetric fission.

This work analyzes *R. sphaeroides* DalA (RSP_1200), a previously uncharacterized protein that was postulated to play a role in cell envelope processes based on the sensitivity of cells containing a transposon insertion in Δ*dalA* to chemicals that target this subcellular compartment and their ability to secrete lipids into the extracellular medium (30). Our findings provide new insight into the composition and dynamic repositioning of multi-protein complexes responsible for *R. sphaeroides* growth and cell division. They also highlight some differences in cell envelope remodeling from what is reported in other bacteria that divide by symmetric fission. This, plus the conservation of DalA in other bacteria, predicts that insights gained from studying the *R. sphaeroides* protein can shed light on cell elongation and division in other species.

### Different cell division proteins in *R. sphaeroides* and *E. coli*

The covalent interaction between the *E. coli* OM lipoprotein Lpp and PG is important for envelope function and stability (53 - 55). In addition, other OM proteins (Pal, OmpA) can non-covalently bind the PG (56 - 58). However, genes encoding homologs of Lpp and other OM lipoproteins are absent in most *Alphaproteobacteria*, including *R. sphaeroides,* suggesting the presence of different systems or proteins in these bacteria. Indeed, several newly characterized OM proteins have been reported to function in envelope stability and covalently bind PG in *Brucella abortus* (36, 59), another member of the *Alphaproteobacteria*.

We find that *R. sphaeroides* DalA is an OM lipoprotein which can non-covalently bind PG. In addition, we show that DalA immunoprecipitates with several PG assembly enzymes, as well as the divisome proteins Pal and FtsZ. Our observations lead us to propose that DalA is a newly identified OM lipoprotein and that this protein plays a role in PG remodeling during cell division.

### DalA is required for normal cell division

We found that the position of DalA in the envelope was dynamic and cell cycle dependent. DalA is positioned at the midcell as the cell cycle progresses before it moved to the septum until the complete septation of daughter cells. We also found that DalA immunoprecipitates and colocalizes with Pal, an OM lipoprotein known to be a key divisome subunit in other bacteria (10, 39, 42, 43, 60). These features, plus the co-immunoprecipitation of DalA with FtsZ and the position of both proteins in Z-rings at the septum, led us to propose that this OM lipoprotein is part of the *R. sphaeroides* divisome.

As predicted by the role of DalA in the divisome, a mutant lacking this protein exhibits a defect during division as seen by the increased percentage of mutant cells that are involved in division compared to a WT counterpart. We also found the presence of OM protrusions at the midcell of the Δ*dalA* mutant, providing an explanation for the previously reported production of extracellular lipids and vesicles in cells lacking DalA (30). In other Gram-negative bacteria, mutants of the *tol-pal* subunits of the divisome machinery are reported to release lipids and form OM protrusions (10, 39). Thus, it is not surprising to find that Δ*dalA* mutants form OM protrusions if DalA is part of a multi-protein complex with Pal and other PG-binding/remodeling proteins. We were able to generate a *R. sphaeroides* Δ*pal* mutant, despite the fact that this gene is reported to be essential in *Alphaproteobacteria* belonging to the *Caulobacterales* and *Rhizobiales* clades (39, 45), suggesting there are differences in the role of this divisome component in individual organisms.

Pal binding to PG helps coordinate the invagination of envelope membranes during division in *E. coli* (42, 43), so it is possible that DalA plays a similar role in *R. sphaeroides*. Differences in the response of the Δ*pal* and Δ*dalA* mutants to mecillinam, in the morphological phenotypes of these mutants, and opposed effects in the level of tri-tri cross-links in the PG predict that Pal and DalA have some distinct functions in the divisome. To date, we have been unable to generate a *R. sphaeroides* Δ*pal*Δ*dalA* double mutant*,* suggesting they represent a synthetic lethal pair that is needed for cell division. In the future, it will be interesting to test how reductions in one or both of these *R. sphaeroides* lipoproteins impact growth or cell division.

### DalA functions in PG remodeling

We found that DalA colocalizes with the site of incorporation of the FDAA HADA in growing cells, suggesting that this protein is close to the site of FDAA incorporation by PG transpeptidases (61, 62). We also found that FDAA incorporation is altered in a Δ*dalA* strain, particularly at the midcell where DalA is known to be positioned, suggesting that this apparent change in PG assembly may be associated with the observed delay in cell division by this mutant. These observations, plus the immunoprecipitation of DalA with several predicted PG LDTs (63), provide evidence for a functional interaction between this OM lipoprotein and the enzymes that form PG cross-links in *R. sphaeroides*. It is known that *R. sphaeroides* has a relatively high PG cross-link content compared to *E. coli* (64) and that mDAP-mDAP cross-links are formed exclusively by LDTs. This could be taken as evidence for a major role of LDTs in *R. sphaeroides*. In support of this hypothesis, we found that a mutant lacking either the predicted LDT RSP_1199 or the Pal lipoprotein has a similar growth phenotype (Fig. 6A) and increased sensitivity to PG active agents that impact cross-link formation (aztreonam). Of the strains tested, only the Δ*RSP_1199* and Δ*dalA* strains showed increased resistance to mecillinam, a compound known to inhibit PG transpeptidase activity. While a specific target(s) for mecillinam in *R. sphaeroides* is not known, previous work on *E. coli* showed that modifications in PG cross-link content can lead to resistance to this antibiotic (65, 66).

Indeed, the analysis of PG composition demonstrated that a loss of *dalA* decreased the levels of tri-tri (mDAP-mDAP) cross-links. Since these cross-links are generated by LDTs, it is likely DalA plays an important role in LDT function. Furthermore, we also found a decrease in the level of a tri-lys-arg fragment in the DalA mutant, a fragment that is composed of a PG residue mDAP cross-linked to residues (lys-arg) from OM-bound protein Lpp in *E. coli* (53). While PG-OM protein cross-links are known to be generated by LDTs (36, 67), the OM protein(s) covalently bound to PG in *R. sphaeroides* are currently unknown. It is known that covalent links between the OM and PG are important for cell envelope stability and that decreased OM-PG crosslinking led to vesicle formation (44, 68). While it is possible that OM-PG covalent interactions like those in *B. abortus* (36) exist in *R. sphaeroides*, additional investigation is needed to understand how the loss of DalA alters LDT activity. In this regard, the co-precipitation of DalA and RSP_1199, the sensitivity, and the increased mecillinam resistance observed in Δ*RSP_1199* and Δ*dalA* mutants suggest these two proteins play a cooperative role in PG assembly.

Over the past decade, many lipoproteins have been shown to interact actively with PG enzymes, either as scaffold for assembly of enzyme complexes (69) or activators of PG hydrolase proteins like *E. coli* NlpD or Lpo (11, 70 - 74). However, many of these lipoproteins, including LpoA and LpoB, have no known homologs in *R. sphaeroides*. Thus, it is possible that DalA serves a similar role as a scaffold or activator of PG LDTs during the cell cycle, possibly at the septum during cell division.

### DalA and envelope proteins needed for elongation and division by *R. sphaeroides*

In rod-shaped bacteria like *R. sphaeroides*, the enzymes for PG synthesis are in close proximity with the MreB, FtsZ, Pal, and other elongasome or divisome subunits (47, 75 - 79). During elongation of rod-shaped bacteria that divide by symmetric fission, like *E. coli* or *Bacillus subtilis*, PG synthesis occurs in a so-called dispersed manner, along the longitudinal side of the cells using enzymes within the elongasome then at the septum using enzymes contained in the divisome (14, 17, 24, 80).

**Fig 9:**
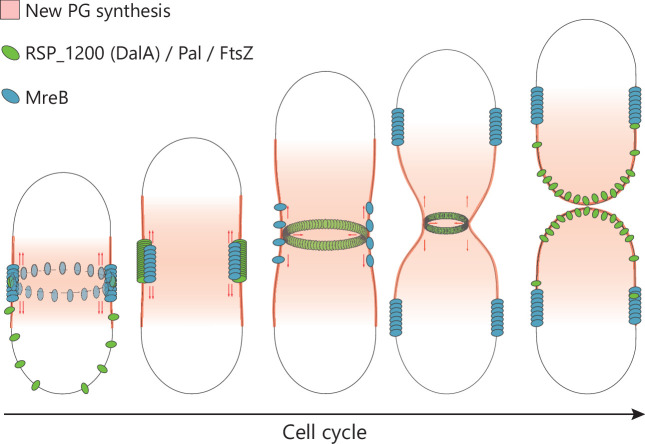
Model for *R. sphaeroides* divisome localization during cell cycle. Model representing the position of DalA, the divisome (green), and MreB (blue) during the cell cycle, along with the insertion of new PG chains (red).

Changes in the subcellular position of MreB and FtsZ in *R. sphaeroides* in other studies (25, 29, 35, 81) plus ours (Fig. S9) show that, unlike some other well-studied bacteria, MreB and FtsZ colocalize at midcell early in the cell cycle. When membrane constriction for cell division starts, MreB moves to the future midcell sites of daughter cells, while the divisome proteins FtsZ, Pal, and DalA remain at the septum, presumably to ensure proper septum constriction and formation of progeny. The continued presence of MreB at the *R. sphaeroides* midcell is both atypical and appears independent of FtsZ, unlike the case in *E. coli* (82). These findings agree with the observed pattern of longitudinal PG synthesis, with the midcell being the major site of new PG synthesis (29). The proposed positioning of PG assembly, plus elongasome and divisome components at the midcell during this part of the cell cycle, suggests that there might be shared or even transient interactions of these multi-protein complexes that have not been reported in other rod-shaped bacteria (Fig. 9). Considering these observations, it might not be appropriate to describe separate elongasome and divisome complexes in organisms like *R. sphaeroides*.

In this regard, it is tempting to propose that an OM lipoprotein like *R. sphaeroides* DalA plays an important role in coordinating the interaction of these multi-protein complexes in this and other *Alphaproteobacteria* during division. Such a role for DalA would support the hypothesis that PG assembly depends highly on the position and composition of cell envelope protein complexes (83). In the future, it will be interesting to determine what processes and systems position these different envelope complexes during the cell cycle.

### DalA homologs are found in other *Rhodobacterales*

Given the properties of DalA revealed from our studies, it is important to note that a similar pattern of FDAA incorporation into PG has been observed in *R. sphaeroides* and *R. capsulatus,* another member of the *Rhodobacterales* clade of the *Alphaproteobacteria* (Fig. S10). Based on this and the role of DalA in cell division predicted by our results, we searched for homologs of this protein in the National Center for Biotechnology Information (NCBI) database. This analysis predicts the presence of DalA homologs in ≈18% of some 450 unique *Alphaproteobacteria* reference genomes. Among the *Alphaproteobacteria*, the fraction of the genomes that contain a DalA homolog is variable, ranging from 93% of the *Rhodobacterales* genomes to 3% of the *Hyphomicrobiaceae* (Fig. S11). Outside of *Alphaproteobacteria*, DalA homologs are found in the genomes of *Terrabacteria* (*Cyanobacteria*, *Actinobacteria,* and *Actinomycetota*), including pathogens such as *Mycobacterium avium*. Our analysis of *Alphaproteobacteria* reference genomes predicts that DalA homologs are found in *Rhodobacteraceae* (*Paracoccus, Rhodobacter,* and *Rhodovulum*) including *R. capsulatus* and *Roseobacteraceae* (*Roseobacter*), species known to divide by symmetric fission (84). In contrast, DalA homologs are not predicted to be present in *Alphaproteobacteria* clades that do not divide by symmetric fission (*Caulobacterales* or *Rhizobiales*). Thus, it will be interesting to determine if DalA homologs are limited to and play similar roles in other bacteria that divide by binary fission.

In conclusion, this study has increased our knowledge on cell envelope growth and division in *R. sphaeroides* and possibly other *Alphaproteobacteria*. By combining genetic, genomic, and microscopic analyses, we showed that the previously uncharacterized OM lipoprotein DalA non-covalently binds PG and plays an important role during cell division in *R. sphaeroides.* We propose that DalA is a subunit of the divisome and that it acts as a scaffold or activator for LDTs during the cell cycle. Our findings also provide testable hypotheses to analyze the role of DalA and its homologs in the growth and function of the cell envelope in *R. sphaeroides* and other bacteria.

## MATERIALS AND METHODS

### Bacterial strains and growth conditions

*R. sphaeroides* strains (Table S1) were grown in Sistrom (SIS) minimal medium (85). As noted in the table, we designate WT as the strain *R. sphaeroides* 2.4.1 with the previously described deletion Δ*RSP_0382* (86), leading to bacteria cells that are incapable of producing poly-β-hydroxybutyrate granules in the cytoplasm, storage compounds that can be used during nutritional deprivation. This mutation does not have any effect on growth or antibiotic sensitivity in our growth conditions (30). This choice was made to avoid bacteria segmentation issues due to the high contrast of these granules in brightfield microscopy. Unless specified, cultures of 10 mL were grown in 125 mL flasks at 30°C with shaking at 200 rpm, until cells reached mid-exponential growth. *E. coli* strains (Table S1) were grown at 37°C in Luria-Bertani medium. As needed, media were supplemented with 50 µg/mL kanamycin and/or 10 µM isopropyl β-d-1-thiogalactopyranoside (IPTG). Cell densities were measured with a Shimadzu spectrophotometer at 600 nm, and growth curves were made by measuring and pooling OD_600nm_ of four independent cultures grown in TECAN M1000 plate reader at 30°C.

### Strain construction

All strains, primers, and plasmids used in this study are listed in Table S1. Construction of plasmids was performed by PCR amplification from *R. sphaeroides* genomic DNA using Herculase II Fusion DNA Polymerase (Agilent, Santa Clara, CA). PCR products were assembled into PCR linearized pIND5 or pk18*mobsacB* vectors by Gibson Assembly (New England BioLabs, Ipswich, MA) and transformed into DH5α cells (New England BioLabs). Transformants were screened by colony PCR and sequenced to confirm there were no mutations in coding regions. Plasmids were mobilized into *R. sphaeroides* via conjugal mating with *E. coli* S17-1 (87). Colony PCR of Kan^R^ *R. sphaeroides* colonies was used to confirm successful mobilization of plasmids. *R. sphaeroides* genomic insertion on the chromosome was constructed by allelic exchange using the suicide vector pk18*mobsacB* as described previously (28). Gene insertions were confirmed by colony PCR of chromosomal loci and sequencing of genomic DNA with gene-specific primers (Table S1).

### Fractionation and proteome analysis

Three-liter SIS cultures were made in Povitsky bottle aerated by a gas mixer (69% N_2_, 30% O_2_, and 1% CO_2_) and a stir bar until late exponential phase (OD_600nm_ ≈0.8). Cells were harvested, resuspended in a MOPS 20 mM/MgCl_2_ 1 mM pH 7.5 solution with Halt protease inhibitor cocktail (Thermo Fisher Scientific, Waltham, MA), and broken two times by French Press at 1,300 psi. After a centrifugation at 11,000 × *g* for 15 min, supernatant was submitted to a first ultracentrifugation in a rotor 70.1ti (Beckman, Pasadena, CA) at 55 krpm for 2 h at 4°C. The membrane pellets were recovered with a homogenizer in MOPS and put on top of an ultracentrifuge tube filled as follows with a sucrose gradient: from bottom, 2 mL 72% sucrose, 2 mL 60%, 2 mL 50%, 2 mL 40%, and 2 mL 20%. Tubes were centrifuged during 16 h at 55 krpm in rotor 70.1ti at 4°C. Different fractions were recovered with the IM on top and the OM at the bottom, then submitted to the University of Wisconsin-Madison MS core for proteome analysis. Briefly, proteins were extracted, digested, and analyzed by Orbitrap Fusion Lumos Tribrid platform. Lumos-acquired MS/MS files were searched using Proteome Discoverer Sequest HT search engine against Uniprot *R. sphaeroides* reference database. Normalization was performed on total peptide amount and scaling on all average.

### Protein purification and co-immunoprecipitation

To purify DalA and limit *R. sphaeroides* protein contaminants, *E. coli* strains carrying plasmid constructions *dalA-mCherry/myc* IPTG inducible were grown with shaking in 2 L flask filled with 250 mL Luria-Bertani broth (LB) with kanamycin 50 µg/mL at 30°C. After 1 h post inoculation, IPTG was added to 50 µM and let grown overnight. Cells were harvested and resuspended in a lysis buffer (Tris 20 mM, NaCl 500 mM, Triton X-100 0.5%, glycerol 10%, pH 7.5). Cells were sonicated on ice. Cells were centrifuged during 20 min at 28,000 × *g* in a JA-20 rotor (Beckman, Pasadena, CA) at 4°C. A 30 µL of nanotrap affinity beads (Chromotek, Rosemont, IL) coupled with antibodies directed either against red fluorescent protein (RFP; for mCherry constructions) or myc tag was equilibrated in a 1.5-mL tube. And 600 µL of obtained cell lysate mixed 1/1 with dilution buffer (Tris 10 mM, NaCl 25 mM, EDTA 0.5 mM, Triton X-100 0.05%, pH 7.4) was added to the beads and let tumbling during 4 h at 4°C. Beads were pulled down at 2,500 × *g* during 5 min at 4°C then washed five times with the dilution buffer. Proteins were eluted by acidic method. Beads are resuspended at 55°C with 70 µL acid solution (200 mM glycine, 200 mM NaCl, pH 2.5) for 1 min by pipetting in a spin column and then centrifuged at 1,500 × *g* during 1 min in a tube with 7 µL of a neutralizing solution (Tris 1 M pH 10.4).

For the co-immunoprecipitation of DalA, 500 mL of the *R. sphaeroides* strain *dalA-ng* grown in SIS medium was used. As a control, a WT strain carrying a plasmid expressing only the *neongreen* gene was also used to identify possible non-specific interactions. The protocol was similar to protein purifications, using NG nanotrap (Chromotek). To elute the proteins, beads were heated with SDS solution. The elutions were submitted to the University of Wisconsin-Madison MS core for protein analysis. Briefly, proteins in the samples were precipitated (10% TCA/62.5% acetone, 30 min on ice) and centrifuged. Pellets were re-solubilized, denatured, and alkylated. Finally, proteins were digested by a mix trypsin/LysC. Peptides were analyzed by nano LC-MS/MS using the Agilent 1100 nanoflow system connected to hybrid linear ion trap-orbitrap mass spectrometer (LTQ-Orbitrap Elite, Thermo Fisher Scientific). MS/MS files were used to search against Uniprot *R. sphaeroides* reference database. Peptide identifications were accepted if they could be established at greater than 78.0% probability to achieve a false discovery rate (FDR) less than 1.0% by the Scaffold Local FDR algorithm. Protein identifications were accepted if they could be established at greater than 96.0% probability to achieve an FDR less than 1.0% and contained at least two identified peptides. Protein probabilities were assigned by the Protein Prophet algorithm (88). Proteins that contained similar peptides and could not be differentiated based on MS/MS analysis alone were grouped to satisfy the principles of parsimony. Proteins sharing significant peptide evidence were grouped into clusters.

### Peptidoglycan purification

In a similar manner as the purification on beads, 500 mL *R. sphaeroides* WT cultures were harvested and sonicated. The lysate was centrifuged, the pellet containing the PG was resuspended in 5 mL of a sodium phosphate buffer (25 mM pH 6), boiled, then the same buffer with 8% SDS was added according to reference (89). The sample was centrifuged at 38,000 × *g* during 15 min, then boiled again with SDS. PG pellets were washed four times with the sodium phosphate buffer to remove SDS. The PG was resuspended in PBS and transferred to a 1.5-mL tube and treated with DNase/RNase (20 µg)/α-amylase (20 µg) at 37°C overnight. The resulting PG was pelleted and rinsed two times then treated with protease (50 µg) overnight at 37°C. PG was heated at 80°C to inactivate protease, pelleted and rinsed two times, and finally resuspended with PBS.

### Peptidoglycan-binding assays

For non-covalent binding tests, purified PG from *R. sphaeroides* (≈50 µg) was mixed with purified DalA-myc, DalA-mCherry, or control BSA (≈300 ng) in tube with PBS pH 7.5, 100 µL total volume, and let on ice for 30 min. Samples were centrifuged for 5 min at 21,000 × *g*, and supernatant recovered as the SN fraction. PG pellets were washed with 100 µL of a modified PBS buffer (NaCl 500 mM, Tween-20 0.025%) and centrifuged again. The supernatant obtained was recovered as the wash (W) fraction. PG and control pellets were washed two more times and recovered as pellet (P) fraction. SN and W fractions were evaporated to a suitable volume. All samples were subsequently analyzed by Western blot. For covalent PG-binding tests, similar method as reference (36) was employed. *R. sphaeroides dalA-mCherry* cultures were grown in SIS until exponential growth phase. Cultures were centrifuged and concentrated to OD_600nm_ = 10 in 100 µL PBS. Cells were heated at 85°C for 50 min. Samples were divided in two, 20 µg of fresh lysozyme (Sigma-Aldrich, St. Louis, MO) was added in one of them, and all samples let overnight at 30°C with shaking. DNase was added to the samples for 30 min at 37°C, then subsequently analyzed by Western blot.

### Peptidoglycan composition analysis

Purified PG samples were digested with mutanolysin to cleave β-N-acetylmuramyl-(1→4)-N-acetylglucosamine linkages before reducing them with sodium borohydride. PG fragments were analyzed using an Acquity UPLC H-Class PLUS with PDA Detector and QDa Detector. Samples were run on a linear gradient of 3.0% acetonitrile with 0.1% formic acid to 11.5% acetonitrile with 0.1% formic acid over 10 min at a flow rate of 0.5 mL/min at 42°C using electrospray ionization in positive ion mode. Fragment quantification and identification were done using MassLynx V4.2 software. Fragments were quantified using the area of peaks quantified at 206 nm as a percentage of the total peaks detected between 2 and 8 min.

### Western blot

NuPAGE sample buffer (Invitrogen, Carlsbad, CA) with DTT 50 mM was added to all samples. Samples were heated for 10 min at 95°C, then loaded on pre-cast NuPage 4-12% Bis-Tris gels (Invitrogen), and separated by electrophoresis in MES or MOPS SDS running buffer. Proteins were transferred on 0.45 µm polyvinylidene difluoride (PVDF) membranes (Invitrogen) at constant amperage (250 mA) for 1 h. Membranes were blocked for 2 hr in Tris saline buffer Tween-20 0.05% (TBST) with milk 5%. Primary polyclonal anti myc-tag antibodies (1/20,000, Abcam, United Kingdom), anti-BSA (1/10,000, Fisher, Hampton, NH), or monoclonal anti-RFP (1/3,000, Chromotek) were added in TBST milk 5% and let overnight at 4°C. Blots were washed with TBST milk three times. Secondary antibodies coupled with horseradish peroxidase (1/3,000 anti-mouse for anti-RFP, 1/10,000 anti-rabbit for others) were added in TBST milk 5% during 1 h at room temperature. After several washes with TBST, signal was detected using SuperSignal West PicoPlus (Thermo Fisher Scientific) and imaged with the Aplegen Omega Lum C.

### Microscopy

All strains were grown as described until exponential growth unless indicated. An amount of 1 µL of culture was immobilized on a glass coverslip under a thin 1.5% agarose pad. For time-lapse studies, a closed Ibidi chamber was used, and SIS medium was added to the pad, pictures were taken either every 45 min or 10 min. When needed, HADA (Tocris Bioscience, United Kingdom) was added at 100 µM during 8 min before washing three times to remove unstained dye then visualized immediately after. For division or blebs visualization, FM4-64 (Setareh Biotech, Eugene, OR) was added at 10 µg/mL before visualization. Microscopy images were taken using an EVOS FL Auto 2 (Invitrogen), equipped with an Olympus 100X oil immersion PLAN Apochromat objective (NA 1.40). EVOS light cubes DAPI, GFP, and Texas Red were used to detect the different fluorescence signals in this study. Structured internal microscopy pictures were taken with a Nikon Eclipse Ti-E N-SIM microscope equipped with an Andor iXion 897 EMCCD camera in 3D-SIM mode. Volumetric rendering was done with plugin ClearVolume in FIJI (90). For helium ion microscopy, cells in exponential growth phase were fixed in fresh PBS solution with glutaraldehyde 2.5% for 2 h. Cells were then carefully washed three times with PBS for 15 min each. A gradual dehydration in ethanol was done, putting cells for 30 min each time successively in 25%, 33%, 50%, and 75% ethanol then 3 × 30 min in 100% ethanol. Centrifugations were conducted at very low speed. The cells were then transferred to the critical point dryer (CPD) (Tousimis, Rockville, MD, Autosamdri-815) and processed according to an automated CPD scheme, with CO_2_ as a transitional fluid. The samples were then mounted on standard carbon tape-covered aluminum scanning electron microscopy (SEM) stubs (Ted Pella, Redding, CA, USA), and sputter coated with carbon. The samples were imaged with a high-resolution Orion Helium ion microscope, (Zeiss, Peabody, MA, USA) at 30 keV.

### Image analysis

All images were analyzed using software FIJI (91) and the plugin MicrobeJ v5.13n (92) with the same parameters for all pictures. To facilitate bacteria detection, all brightfield pictures were treated as follows: invert, bandpass filter (large filter, 40 pixels; small filter, 2 pixels), background subtraction (rolling ball radius = 20 pixels), and contrast enhancing with normalization (0.1%). Fluorescence pictures were only treated with background subtraction (radius = 50 pixels). When needed, image stacks were aligned with plugins Image stabilizer and StackReg (93). Bacteria segmentation was performed on the treated brightfield images using MicrobeJ and verified manually for errors. For each figure, the analysis of at least three independent experiments was pooled together, each containing at least 500 detected bacteria. Fluorescence demographs were obtained on MicrobeJ using a medial profile with the mean orthogonal projection of pixel values along the medial axis as method. Shape, fluorescence, and maxima localization parameters of cells were extracted from MicrobeJ, and figures were generated on RStudio using, in particular, package ggplot2 (94). Statistical analyses were performed on RStudio, using, if not specified otherwise, unpaired two-tailed *t*-tests, with the means calculated for each biological replicate as parameters (95).

### Spot titer assay

*R. sphaeroides* single colonies were grown with shaking at 30°C until late exponential growth phase (OD_600nm_ ≈1); they were diluted to an OD_600nm_ of 0.5 in 200 µL of Sistrom medium in a 96-well plate. An amount of 20 µL of this dilution was then serially diluted seven times into 180 µL Sistrom. Sistrom agar plates were made with the appropriate concentration of antibiotics tested. An amount of 9 µL of the dilution series was spotted, allowed to dry, and then incubated at 30°C for several days. Each antibiotic was tested at least three times on each strain on plate.

## SUPPLEMENTAL MATERIAL

Supplemental material mbio.01203-23-s0001.pdf Supplemental figures and table, with legends and references.

ASM does not own the copyrights to Supplemental Material that may be linked to, or accessed through, an article. The authors have granted ASM a non-exclusive, world-wide license to publish the Supplemental Material files. Please contact the corresponding author directly for reuse.
