# Supplementary material for: A previously uncharacterized divisome-associated lipoprotein, DalA, is needed for normal cell division in Rhodobacterales
Source: mBio. 2023 Jun 30;14(4):e01203-23. doi: 10.1128/mbio.01203-23 (PMC10470522; doi:10.1128/mbio.01203-23)
Supplement: Supplemental material — Supplemental figures and table, with legends and references. [file mbio.01203-23-s0001.pdf]

SUPPLEMENTAL MATERIAL

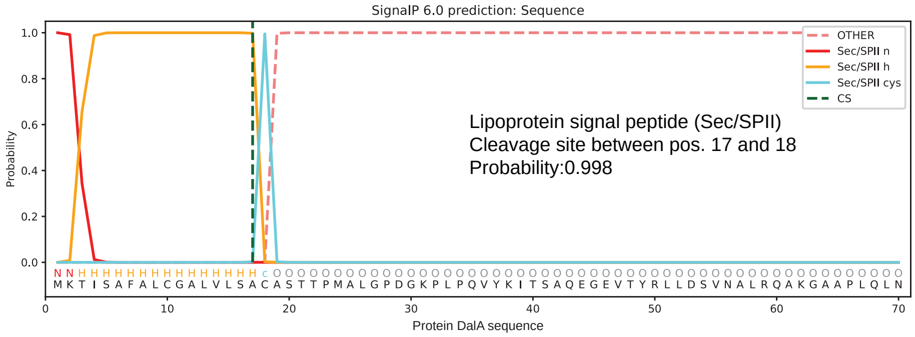

FIG S1 **Predicted lipoprotein signal peptide of DalA.** SignalP 6.0 was used to predict the presence of lipoprotein signal peptide and the cleavage site of protein DalA.

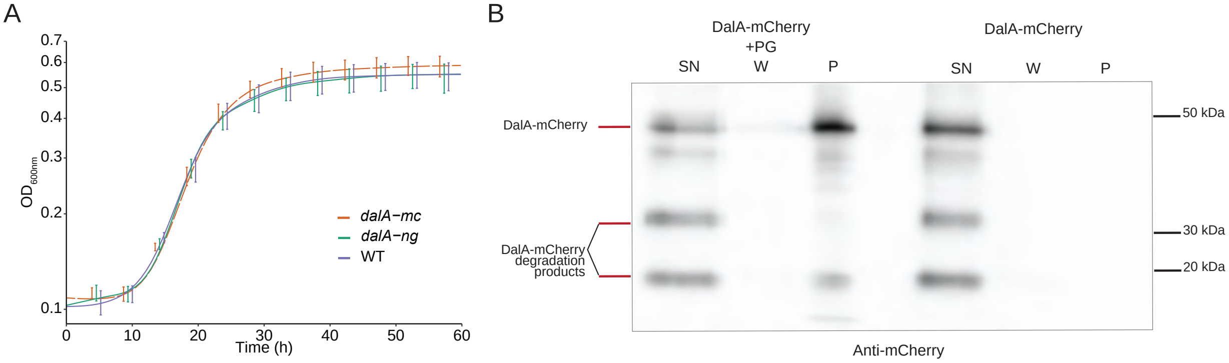

FIG S2 **DalA-mCherry binds PG.** **(A)** Average growth curves of strains *dalA-mCherry* (dashed orange line), *dalA-ng* (green line) and WT (purple line), obtained from 4 independent cultures grown at 30°C. **(B)** Western blot analysis of *in vitro* binding of DalA to PG. DalA-mCherry was incubated with or without PG on ice 30 min then centrifuged. The supernatant fraction was recovered (SN) after a first centrifugation and the PG pellet washed 3 times. The first wash fraction (W) and the pellet (P) containing the PG were recovered and separated by electrophoresis.

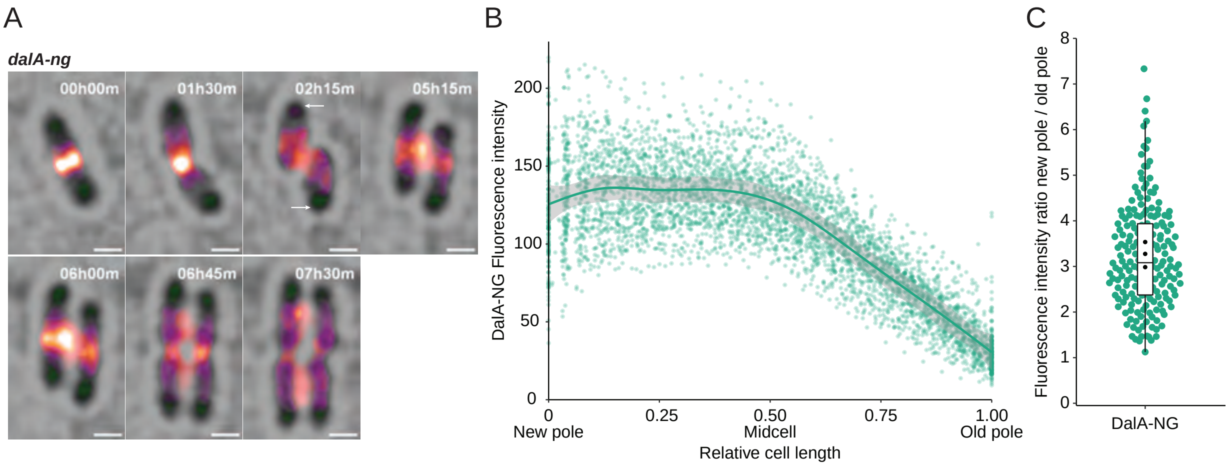

FIG S3 DalA polarity in newly divided cells. (A) Pictures of a time-lapse recording set with a 45 min interval of a dalA-ng cell cycle, the brightfield and DalA-NG fluorescence signal (plasma scale) were merged. As indication, white arrows show old poles after the first division. Scale bars represent 1 µm. **(B)** From the time-lapse experiments on dalA-ng strain, fluorescence intensity profiles of DalA-NG only in newly divided cells (first picture taken showing separation) were measured along the medial axis (n = 194 cells). Cell lengths were normalized and cell polarities were assessed based on the image preceding the daughter cells separation, to display new poles on left (0 on x axis) and old poles on right (1 on x axis). Green dots represent all individual measures, the green line represents the average profile with the standard deviation in grey. **(C)** For each cell, the ratio of DalA mean fluorescence at the new pole divided by the old pole have been calculated (green dots). Limits of the poles on the long axis are determined by the width of the cell divided by two. Results are displayed as a violin plot. Black dots inside the box plot represents the mean of each independent experiment, black bar represents the global median.

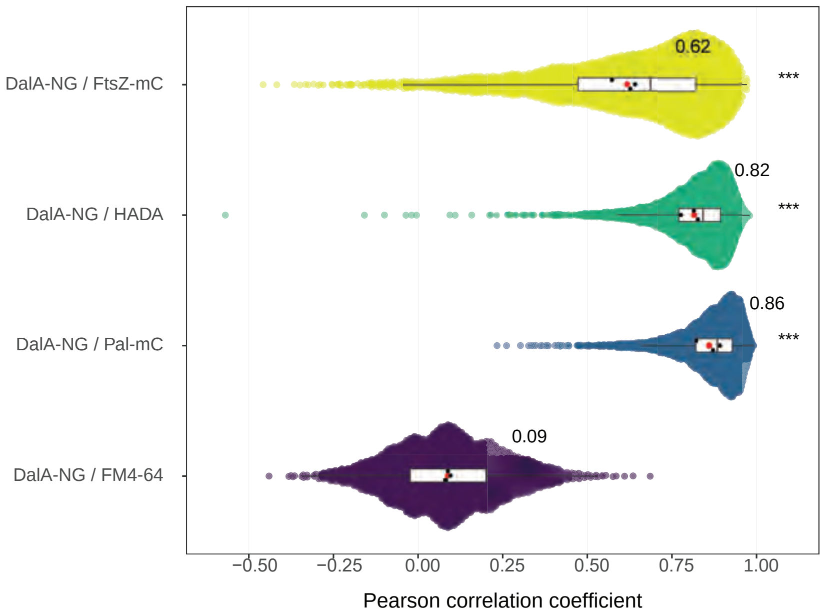

FIG S4 Pearson correlation of fluorescence signals. Pearson correlation coefficients were calculated to quantify the colocalization of different fluorescence signals using MicrobeJ. In *dalA-ng* cells, coefficients were calculated between signals DalA-NG and HADA and as a control between DalA-NG and FM4-64. Coefficients of DalA-NG and Pal-mCherry were calculated in cells containing both proteins. Finally, a strain containing *dalA-ng* and *ftsZ-mCherry* was used to test the correlation between these two proteins. The values range from -1 (anti-correlation) to 1 (perfect correlation, 0 being no correlation) and are represented as violin plots. Black dots represent the mean of each biological replicate, and the red dot is the global mean with the corresponding value as text aside. *P* values were computed from mean values of biological replicates by unpaired two-tailed t-tests with the control DalA-NG/FM4-64 as reference group (***= $p \leq 0.001$).

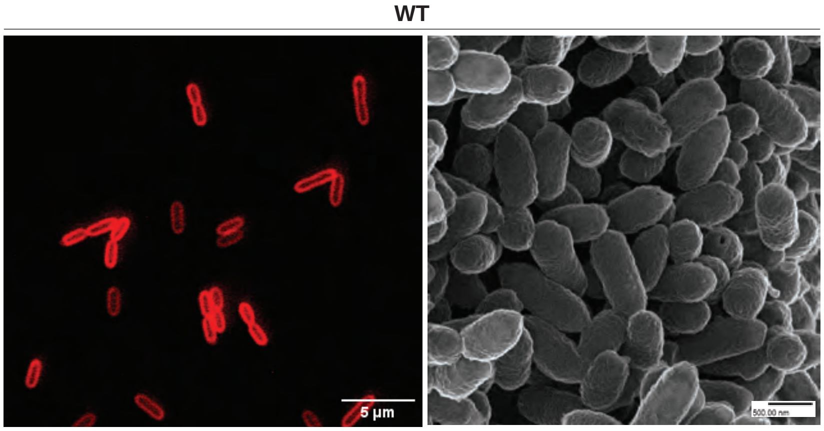

FIG S5 **Membranes microscopy of wild type.** Fluorescence image of wild type (WT) *R. sphaeroides* cells stained with FM4-64 (left), Helium-ion microscopy image of the same strain (right).

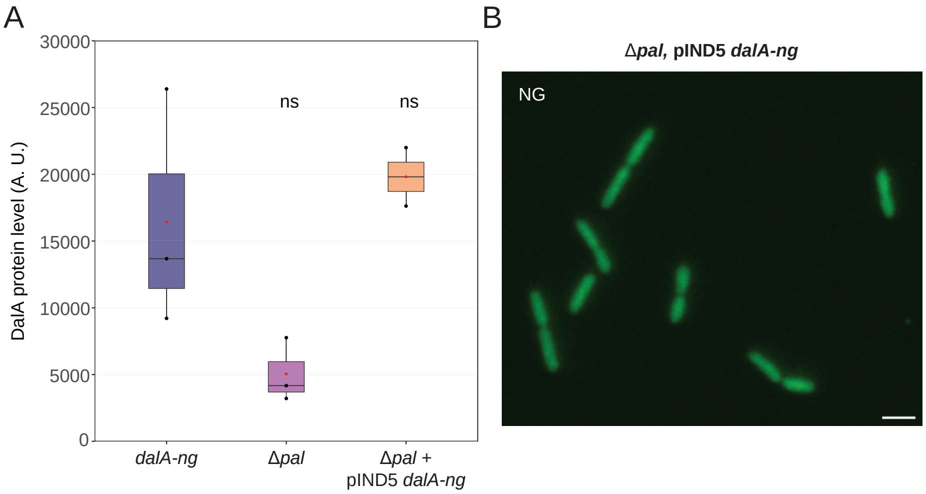

FIG S6 **Δ*pal* impacts DalA levels and localization.** **(A)** Box plot quantification of levels of DalA-NG obtained from western blots with cells *dalA-ng*, Δ*pal,* and Δ*pal*+pIND5 *dalA-ng* (downstream of its native promoter). Black dots represent intensity value from each replicate and red dot is the average. **(B)** Fluorescence microscopy image of Δ*pal* cells containing pIND5 *dalA-ng*. Scale bar represents 2 µm.

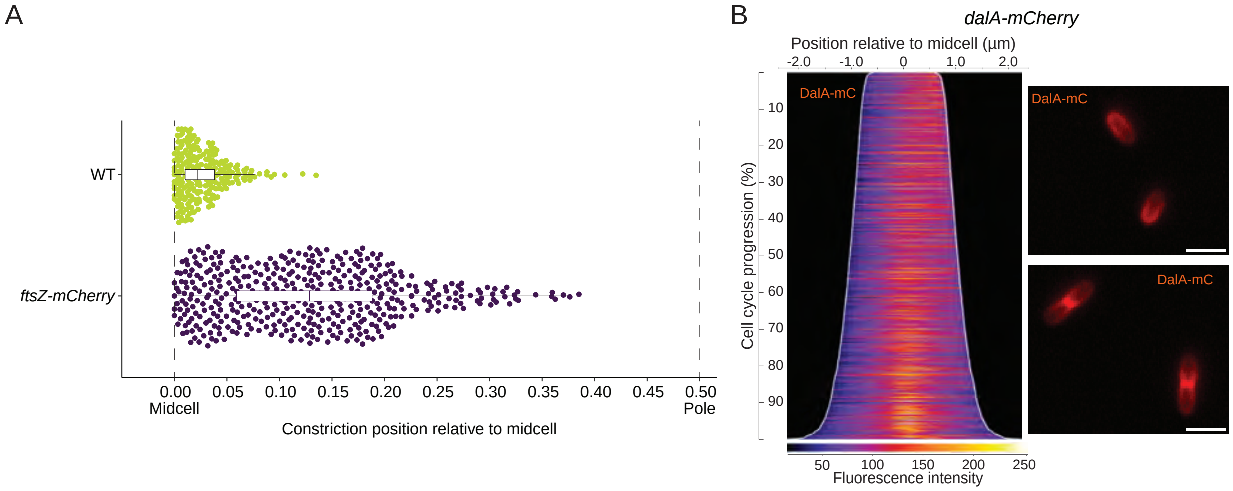

FIG S7 ***ftsZ-mCherry* Z-rings localization and *dalA-mC*.** **(A)** The relative position of membrane constrictions in cells was determined in *ftsZ-mCherry* and WT strains. For WT cells, FM4-64 was used to ensure the presence of membrane constrictions. The distribution of the relative distance of constrictions from midcell is displayed as violin plots, 0 being at the midcell and 0.5 at the pole. **(B)** Micrographs showing DalA-mCherry fluorescence signal in strain *dalA-mCherry* (right), scale bars represent 2 µm. Corresponding demograph (left) of DalA-mCherry fluorescence profiles measured along long axis, cells were sorted longitudinally by length and laterally by pole intensity.

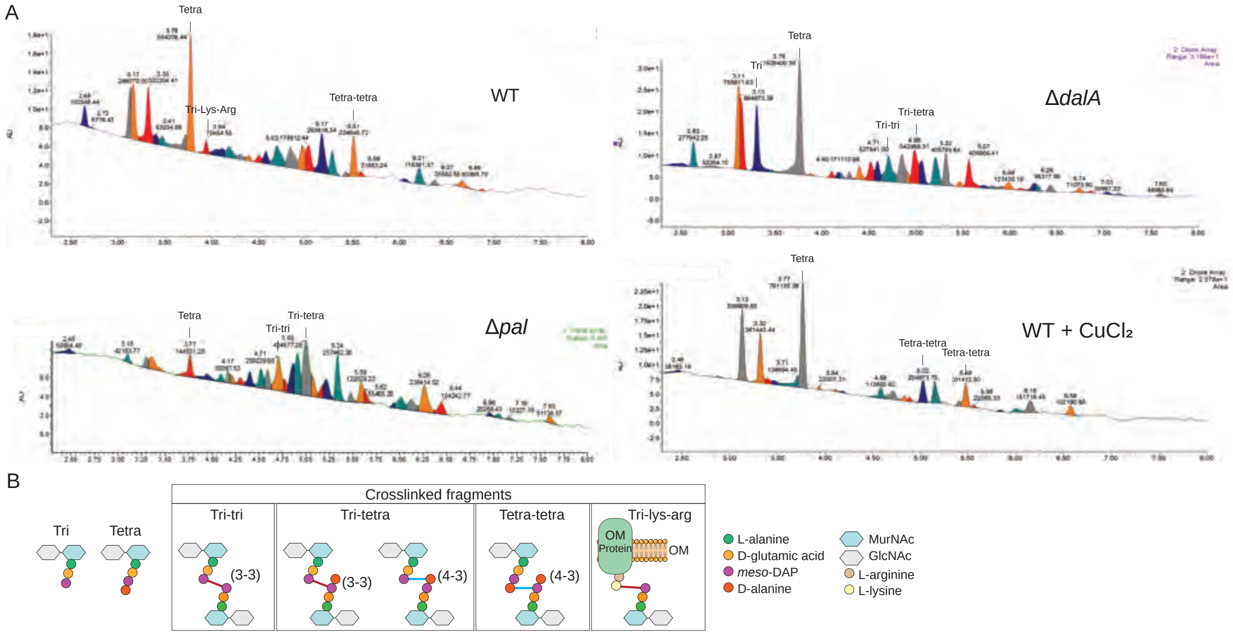

FIG S8 **Muropeptide composition of *R. sphaeroides* strains.** **(A)** HPLC chromatograms of muropeptides of exponential phase cultures of wild type (WT), Δ*dalA*, Δ*pal*, and WT cells grown in presence of $CuCl_2$ (1mM). The identity of some of the representative peaks is annotated on top. **(B)** Graphical representation of muropeptides and the types of crosslinks.

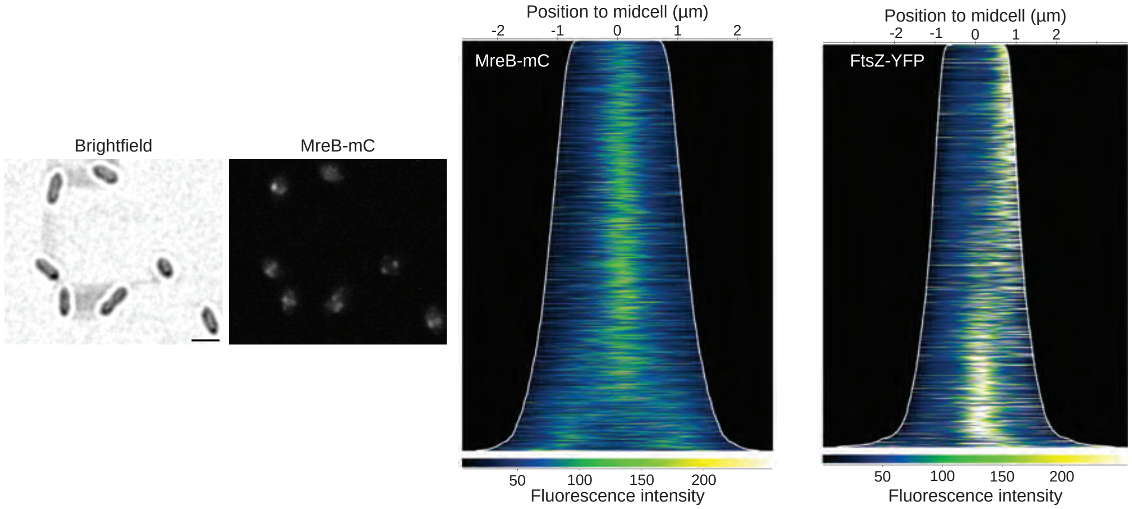

FIG S9 **MreB and FtsZ localization in *R. sphaeroides*.** (Left) Micrographs showing brightfield channel and mCherry-MreB fluorescence of cells containing a *mCherry-mreB* fusion. (Right) Demograph of the mCherry-MreB fluorescence in cells containing a *mCherry-mreB* fusion and FtsZ-YFP fluorescence in cells containing pIND5 *ftsZ-YFP*. For cells containing pIND5 *ftsZ-YFP*, 10µM IPTG was added to cultures to increase expression of the *ftsZ-YFP* fusion. Cell profiles were sorted by length from top to bottom and from left to right to display the most intense pole on right. Scale bar represents 2 µm.

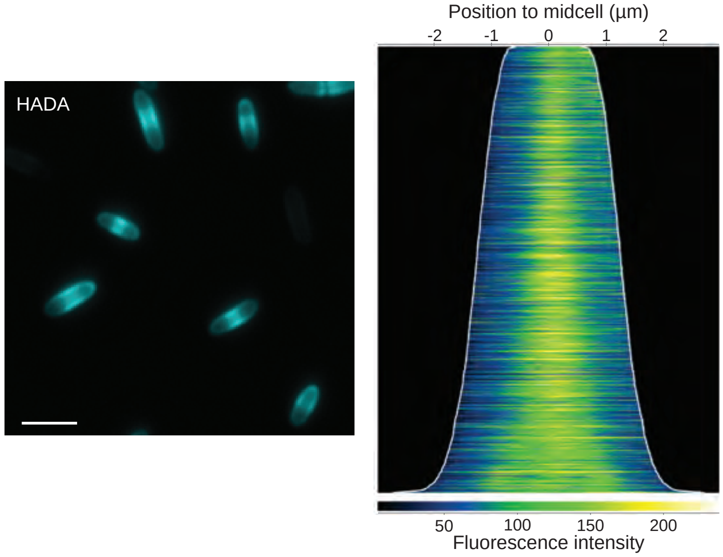

FIG S10 **HADA staining in *R. capsulatus*.** (Left) Micrograph of HADA fluorescence of wild type *R. capsulatus SB1003* and (right) the corresponding demograph with profiles sorted by length from top to bottom and from left to right to display the most intense pole on right. Scale bar represents 2 µm.

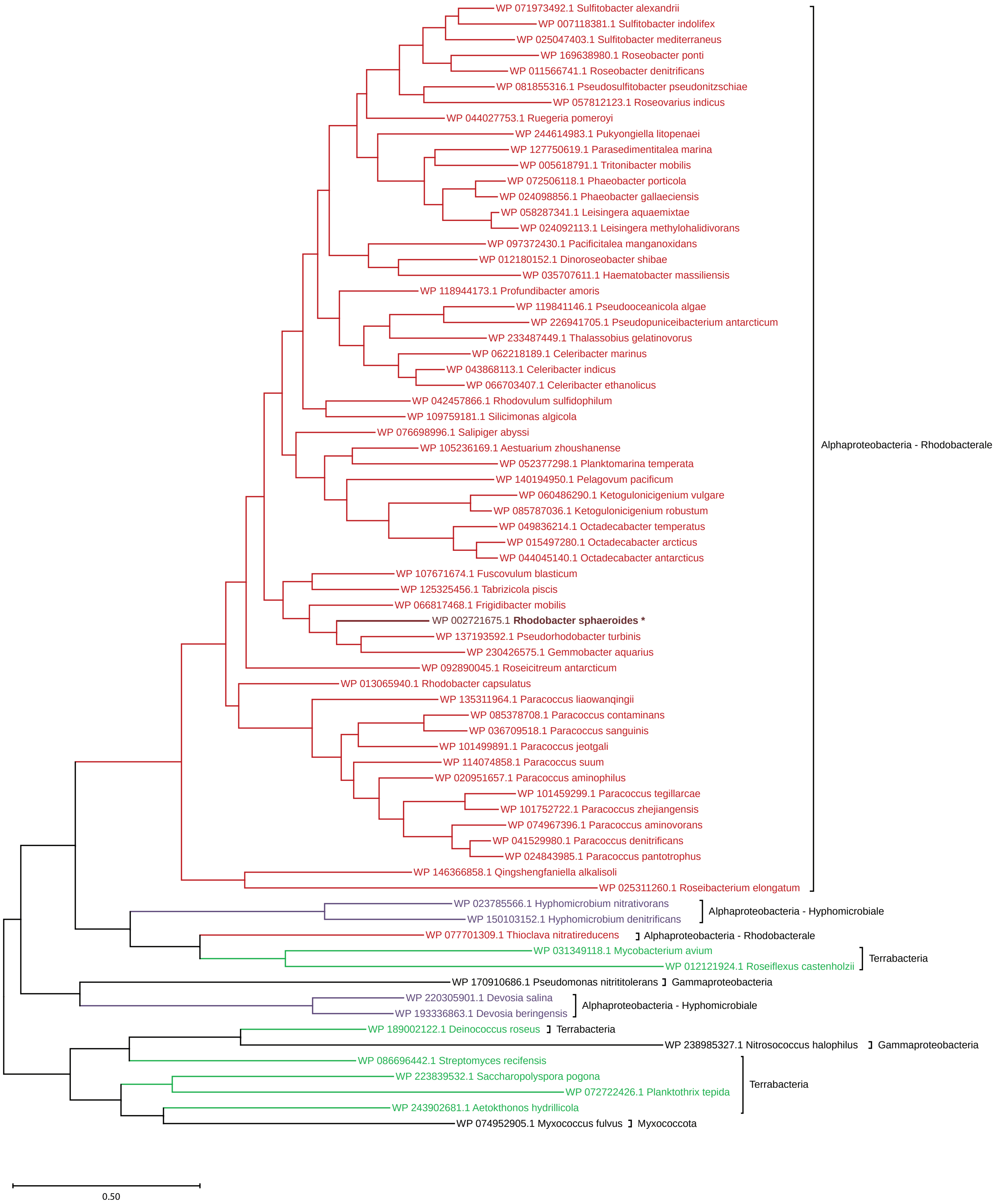

FIG S11 **Phylogenetic tree of DalA homologues.** Homologues amino acid sequences were obtained by Blastp against a database of 450 unique *Alphaproteobacteria* with sequenced genomes. Protein sequences were aligned using MUSCLE and tree was inferred by using the Maximum Likelihood method and JTT matrix-based model. Analyses were conducted in MEGA X software (1)

Table S1: Strains, plasmids and primers used

Strain / named as	Relevant Genotype / Description	Reference	Primers used
WT*	*R. sphaeroides 2.4.1* with Δ*RSP_0382* (see method for explanation)	*R. sphaeroides 2.4.1* (2)	
TD332 / Δ*dalA*	WT* with deletion of gene *dalA*	(3)	
TDF88 / *dalA-ng*	WT* with *dalA-neonGreen* in place of genomic *dalA*	This work	81: TCGAGCTCGGTACCCGGGGATCCAAGATATTACCTCGTTACTCCG<br>82: AGTCTTCATGTGTCTTCCACCCACAAGG<br>83: GGAAGACACATGAAGACTATCTCCGCGTTCG<br>84: AGATGCGGGTTAGGATCCTGAGCCGC<br>85: GGATCCTAACCCGCATCTGCCGCG<br>86: AAACGACGGCCAGTGCCAAGCTTACCCTGCTGACCAAAGATTGTC
TDF229 / *dalA-mCherry*	WT* with *dalA-mCherry* in place of genomic *dalA*	This work	289: TGTACAAGTAACCCGCATCTGCCGCGC<br>290: CCTTGCTCACGAGACCCGCCACCGC<br>291: GCGGGTCTCGTGAGCAAGGGCGAGGAGGA<br>292: GGCAGATGCGGGTTACTTGTACAGCTCGTCCATGCCG
TDF317 / *pal-mCherry*	WT* with *pal-mCherry* in place of genomic *pal*	(4)	
TDF209 / *pal-mCherry, dalA-ng*	TDF88 with *RSP_0668(pal)-mCherry* in place of genomic *RSP_0668*	This work	202: CACCCCTGCGCATGCAAGCTTGGCACTG<br>203: CGCCCGAGCGGAATTCGTAATCATGTCATAGCTGTTTCC<br>204: TACGAATTCCGCTCGGGCGGC<br>205: CTTGCATGCGCAGGGGTGGGC<br>206: TGTACAAGTAATGATCCCATGCGCACCCT<br>207: CTTGCTCACGACCCCCGCACCCAT<br>208: GCGGGGGTCGTGAGCAAGGGCGAGGAGGATAACATGG<br>209: CGCATGGGATCATTACTTGTACAGCTCGTCCATGCC
TDF325 / Δ*dalA, pal-mCherry*	TD332 with *pal-mCherry* in place of genomic *pal*	This work	Same as TDF209
TD271 / *dalA-ng,* Δ*pal*	TDF88 with deletion of gene *pal*	This work	202: CACCCCTGCGCATGCAAGCTTGGCACTG<br>203: CGCCCGAGCGGAATTCGTAATCATGTCATAGCTGTTTCC<br>204: TACGAATTCCGCTCGGGCGGC<br>205: CTTGCATGCGCAGGGGTGGGC<br>261: TCTGCGAAGGACATTTCCCTCTCGATGGGTGCGGGGGTCTGA<br>262: CCCCGCACCCATCGAGAGGGAAATGTCCTTCGCAGATCGGGCG
TD335	WT* with deletion of gene *pal*	This work	Same as TD271
TDF259 / *dalA-ng, ftsZ-mCherry*	TDF88 with *ftsZ-mCherry* in place of genomic *ftsZ (RSP_2114)*	(4)	
TD178 / Δ*RSP_1199*	WT*with deletion of gene *RSP_1199*	This work	188: CATGGTTACGTCCGCTCGTCTCCTTGATCTTGG<br>189: CCGGGGGCCGTAACCATGCAACAGTGAACGG<br>190: TGGTTACGGCCCCCGGCGGG<br>191: AGACGAGCGGACGTAACCATGCAACAGTGAAC
TD179 / Δ*RSP_1201*	WT* with deletion of gene *RSP_1201*	This work	198: GCCTGCCGGTTCACTGCCCGACATGTTTTTTATTC<br>199: CAGTGAACCGCGCCCGCCGC<br>200: CGGGCGCGGTTCACTGCCCGACATGTTTTTTATTC<br>201: GCAGTGAACCGGCAGGCGCCG
TD231 / Δ*RSP_0243*	WT* with deletion of gene *RSP_0243*	This work	269: TCCTCCGCGGCATGCAAGCTTGGCAC<br>270: GCGCGGGCACGAATTCGTAATCATGTCATAGCTGTTTCC<br>271: CGAATTCGTGCCCGCGCTTTCG<br>272: TCCCCGATCATCCGCTGTCCTGCCCC<br>273: GACAGCGGATGATCGGGGATCACCCGTTGC<br>274: TGCATGCCGCGGAGGAGGTGATGACCA
TDF332	TDF271 containing pIND5 *dalA-ng* under its natve promoter control	This work	123: TTAAAGAGGAGAAATTAACAGTCCGGCCGCGGCGGGTC<br>28: AGTCCAAGCTCAGCTAATTAAGCTTTTAGGATCCTGAGCCGCTTC
TDF319	*Rhodobacter capsulatus* SB1003 WT		

TDF164 / *mCherry-mreB*	TDF88 with *mCherry-mreB*	(4)	
TDF270	TDF229 with pIND4 *ftsZ-YFP*	(5)	

Escherichia coli strains

DH5α		(New England Biolabs C2987H)	
S17-1	Strain *λpir* capable of mobilizing pIND5 and pk18*mobsacB* into *R. sphaeroides*	(6)	
TDF144	DH5α with pIND5 *dalA-myc*	This work	123: TTAAAGAGGAGAAATTAACAGTCCGGCCGCGGCGGGTC<br>164: TCACAGGTCCTCCTCCGAGATCAGCTTCTGCTCGAGACCCGCCACCGC<br>165: AGTCCAAGCTCAGCTAATTATCACAGGTCCTCCTCCGAGA<br>166: CTAGAATTAAAGAGGAGAAATTAAATGAAGACTATCTCCGCGTTCG
TDF328	DH5α with pIND5 *dalA-mCherry*	This work	397: CTGTACAAGTAAAAGCTTAATTAGCTGAGCTTGGACTCCTGT<br>398: CCTTGCTCACGGTGGCGACCGGTGGA<br>399: GTCGCCACCGTGAGCAAGGGCGAGGAG<br>400: GCTAATTAAGCTTTTACTTGTACAGCTCGTCCATGCC

Vector plasmids

pIND4	IPTG-inducible vector with Kanamycin resistance	(7)
pIND5	IPTG-inducible vector with modified restriction sites and Kanamycin resistance	(7, 8)
pk18*mobsacB*	Allelic exchange suicide vector with kanamycin resistance	(9)

Supplemental References

1. Kumar S, Stecher G, Li M, Knyaz C, Tamura K. 2018. MEGA X: molecular evolutionary genetics analysis across computing platforms. Molecular biology and evolution 35:1547.

2. Mackenzie C, Choudhary M, Larimer FW, Predki PF, Stilwagen S, Armitage JP, Barber RD, Donohue TJ, Hosler JP, Newman JE. 2001. The home stretch, a first analysis of the nearly completed genome of Rhodobacter sphaeroides 2.4. 1. Photosynthesis research 70:19-41.

3. Lemmer KC, Zhang W, Langer SJ, Dohnalkova AC, Hu D, Lemke RA, Piotrowski JS, Orr G, Noguera DR, Donohue TJ. 2017. Mutations That Alter the Bacterial Cell Envelope Increase Lipid Production. mBio 8:1-15.

4. Lakey BD, Alberge F, Parrell D, Wright E, Noguera DR, Donohue TJ. 2023. The role of CenKR in the coordination of Rhodobacter sphaeroides cell elongation and division. mBio (in press).

5. Chiu SW, Roberts MA, Leake MC, Armitage JP. 2013. Positioning of chemosensory proteins and FtsZ through the Rhodobacter sphaeroides cell cycle. Mol Microbiol 90:322-37.

6. Simon R, Priefer U, Pühler A. 1983. A broad host range mobilization system for in vivo genetic engineering: transposon mutagenesis in gram negative bacteria. Bio/technology 1:784-791.

7. Ind AC, Porter SL, Brown MT, Byles ED, de Beyer JA, Godfrey SA, Armitage JP. 2009. Inducible-expression plasmid for Rhodobacter sphaeroides and Paracoccus denitrificans. Appl Environ Microbiol 75:6613-5.

8. Nam TW, Ziegelhoffer EC, Lemke RA, Donohue TJ. 2013. Proteins needed to activate a transcriptional response to the reactive oxygen species singlet oxygen. mBio 4:e00541-12.

9. Schäfer A, Tauch A, Jäger W, Kalinowski J, Thierbach G, Pühler A. 1994. Small mobilizable multi-purpose cloning vectors derived from the Escherichia coli plasmids pK18 and pK19: selection of defined deletions in the chromosome of Corynebacterium glutamicum. Gene 145:69-73.
